# Patterns in Protein Flexibility: A Comparison of NMR “Ensembles”, MD Trajectories, and Crystallographic B-Factors

**DOI:** 10.3390/molecules26051484

**Published:** 2021-03-09

**Authors:** Christopher Reinknecht, Anthony Riga, Jasmin Rivera, David A. Snyder

**Affiliations:** Department of Chemistry, College of Science and Health, William Paterson University, 300 Pompton Rd, Wayne, NJ 07470, USA; reinknechtc1@student.wpunj.edu (C.R.); anthony_riga@aol.com (A.R.); jaz_x33@yahoo.com (J.R.)

**Keywords:** Friedman’s test, backbone atom coordinate variances and uncertainties, superimposition

## Abstract

Proteins are molecular machines requiring flexibility to function. Crystallographic B-factors and Molecular Dynamics (MD) simulations both provide insights into protein flexibility on an atomic scale. Nuclear Magnetic Resonance (NMR) lacks a universally accepted analog of the B-factor. However, a lack of convergence in atomic coordinates in an NMR-based structure calculation also suggests atomic mobility. This paper describes a pattern in the coordinate uncertainties of backbone heavy atoms in NMR-derived structural “ensembles” first noted in the development of FindCore2 (previously called Expanded FindCore: DA Snyder, J Grullon, YJ Huang, R Tejero, GT Montelione, *Proteins: Structure, Function, and Bioinformatics* 82 (S2), 219–230) and demonstrates that this pattern exists in coordinate variances across MD trajectories but not in crystallographic B-factors. This either suggests that MD trajectories and NMR “ensembles” capture motional behavior of peptide bond units not captured by B-factors or indicates a deficiency common to force fields used in both NMR and MD calculations.

## 1. Introduction

Large molecules and biomolecules can have a high degree of motional flexibility, affecting their function [1]. Common sources of information about protein flexibility and modes of motion include crystallographic B-factors [2], molecular dynamics (MD) simulations [3], and Nuclear Magnetic Resonance (NMR) spectroscopy, including relaxation measurements [4,5,6,7] and even chemical shift data [8,9].

Each of the above techniques for evaluating protein flexibility yields an incomplete picture of protein dynamics in solution. Crystallographic B-factors are affected by packing and other special features of the crystalline state [10]. In addition, many factors may reduce the intensities of the “reflections” in a protein crystal’s X-ray diffraction pattern, and, hence, elevated crystallographic B-factors that may not solely indicate macromolecular flexibility [11]. The quality of MD simulations is dependent on the quality of the seed structure and force field used, despite recent efforts applying MD simulations to NMR-derived structures [12]. NMR relaxation experiments provide a critical source of data for evaluating individual MD trajectories as well as the force fields and other methodological details of MD simulations [13,14]. The combination of multiple assessments of protein flexibility has proven particularly illuminating [15]. For example, the combination of NMR-relaxation data with MD simulations yields a detailed picture of protein dynamics and motional modes [16].

While lacking a universally accepted analog of the B-factor, the NMR-based structure determination process itself provides insight into protein flexibility. Atoms in loop residues and other flexible regions of a protein typically have fewer long-range “contacts” to atoms in other residues. This paucity of contacts leads to both increased flexibility of loop regions [17,18] as well as poor convergence for loop residue positions in NMR-based structure calculations [19,20,21,22], provided the structure refinement process does not lead to inaccurate rather than imprecise coordinates [23]. Moreover, the primary source of structural restraints in NMR-based structure calculations are NOESY (Nuclear Overhauser Effect Spectroscopy) experiments. Fast motions reduce NOEs while intermediate time scale motion causes line broadening that can interfere with the identification of NOESY cross-peaks. Thus, NMR yields a paucity of restraints for particularly flexible regions of a protein leading to poor convergence in NMR-based structure determination, and coordinate uncertainties in an NMR-derived “ensemble” of structures [24]. While, strictly speaking, such coordinate uncertainties measure the local reproducibility of the NMR-based structural determination process, coordinate uncertainties across NMR ensembles are highly correlated to coordinate variances across MD trajectories [25].

While they can provide key insights into protein flexibility and dynamics, evaluation of uncertainties in protein structure coordinates inferred from NMR data is a non-trivial and non-physical process. Typically, NMR-based structure calculations generate multiple (typically 10–40) models [22]. Such collections of structural models are called “ensembles”. Although NMR ensemble generation can effect Boltzmann sampling [19,20,21], generally NMR ensembles, including those analyzed in this study, are not actually Boltzmann ensembles.

Calculation of coordinate variances requires the superimposition of NMR ensembles. However, inclusion of poorly converged coordinates can bias the superimposition process, reducing the applicability of the resulting coordinate variances [26,27]. Limiting the calculation of an optimal superimposition to a core atom set, determined in a superimposition independent manner using either circular variances of backbone dihedral angles [28] or an interatomic variance matrix [27,29], ensures calculation of optimal superimpositions and, hence, of appropriate coordinate uncertainties. Alternatively, assumptions concerning the distribution of coordinate variances can lead to model-based superimposition methods such as THESEUS, which assumes a multivariate Gaussian distribution of coordinate uncertainties [30,31].

Identification of a core atom set is a critical step in solving two distinct, albeit related, problems. Not only does identification of a core atom set an important step in calculating coordinate uncertainties via superimposition, but such a core atom or residue sets also convey in which regions the NMR-based structure calculation process has converged [22]. Since these two problems are different, their optimal solutions may differ slightly. For example, application of the FindCore method, which identifies core atom sets for use in assessing the precision of NMR ensembles, to the distinct, albeit related problem of identifying well-converged core atom sets for CASP10 [32,33], required extension of the FindCore method into an approach known as Expanded FindCore [22].

Software used in the CASP10 competition also required any residue with core atoms to have all backbone heavy atoms in the core. The process of modifying Expanded FindCore to meet this requirement revealed carbonyl oxygens from otherwise well-defined residues whose positions were poorly defined in NMR-based structural calculations. Given the relation between coordinate uncertainties in NMR-derived structures and physical flexibility as described above, this discovery raised questions about the high uncertainties (relative to other backbone heavy atoms in the same residue) of those carbonyl oxygens. How common are these relatively uncertain carbonyl oxygens and is this high relative uncertainty an artifact of the NMR-based structure determination process or is it indicative of a pattern in backbone atom flexibilities?

Addressing these questions requires a comparison of NMR ensembles with complementary structural information, such as that obtained from crystallographic data, as well as with MD trajectories that provide insight into protein flexibility. Protein structures obtained by the North East Structure Genomics (NESG; http://www.nesg.org/ accessed on 31 December 2020) consortium facilitated this analysis. The NESG performed crystallization and HSQC (Heteronuclear Single Quantum Coherence Spectroscopy) screening in parallel for robustly expressed protein targets resulted in more than 40 NMR/X-ray crystal structural pairs [34,35].

The analysis presented here demonstrates the persistence of a pattern in coordinate variances across structural “ensembles” obtained using multiple force fields, superimposition techniques, and sampling schemes (i.e., restrained, simulated annealing and similar schemes in NMR structural refinement vs. the unrestrained constant temperature approach used in MD). This persistent pattern does not necessarily occur in Crystallographic B-factors of backbone heavy atoms. That the relatively high uncertainty of carbonyl oxygens persists, in almost all MD trajectories simulated in this study, indicates that the relatively high uncertainty of carbonyl oxygens is not solely an artifact of NMR-based structural determination. The pattern in backbone heavy atom coordinate uncertainties reflects either a physical reality of peptide bond motion not evident in crystallographic data or a shortcoming common to multiple force fields. If the latter explanation is true, the analysis presented here underscores that further improvements in force field parameterization are necessary for better prediction and calculation of a protein structure and dynamics.

## 2. Results and Discussion

**Figure 1:**
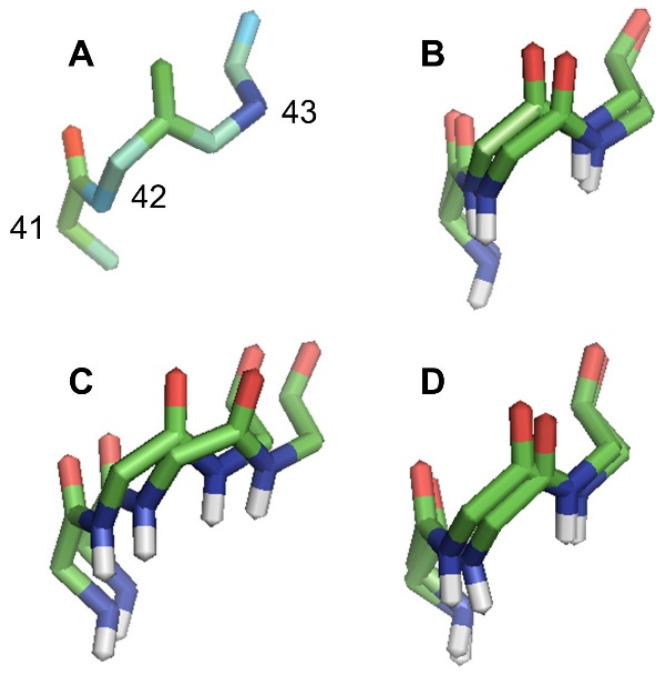
Backbone traces of residues 41–43 from Q8ZRJ2. (**A**) Crystallographic structure (PDB ID 2ES9) colored by a B-factor with blue being low, green being moderate, and red being high. Residue numbers shown in this panel reflect residue numbers in all panels. (**B**) FindCore superimposition of NMR ensemble (PDB ID 2JN8). This superimposition was calculated using a core atom set drawn from all heavy atoms (using all deposited models in the FindCore calculation) and not merely the residues shown. THESEUS superimposition, calculated from the entire MD trajectory using all heavy atoms, of MD trajectories simulated using the AMBER force field, showing snapshots 100 and 1000, at (**C**) 100 K and (**D**) 300 K. In panels (**B**–**D**), carbonyl oxygens are red, amide nitrogens are blue, carbons are green, and amide hydrogens are white. Note the splaying in the carbonyl oxygens in panels (**B**–**D**) and the relatively well superimposed amide nitrogens in panels (**B**) and (**D**). Even in panel (**C**), amide nitrogens are better superimposed than carbonyl oxygens. In general, peptide planes appear to pivot with the amide protons and/or amide nitrogens being relatively immobile with the carbonyl oxygens at the opposite end of the peptide plane being relatively mobile. This pattern is not apparent in the B-factors depicted in panel (**A**).

Figure 1 illustrates how coordinate uncertainty in NMR-derived “ensembles” (panel B) tracks coordinate variance in MD simulations (e.g., at 300 K in panel D). The position of the carbonyl oxygen atom in residue 42 varies both across structural models in the NMR ensemble and over MD trajectories (panels C and D), and this oxygen atom is splayed more than the carbonyl carbon to which it is attached in panels B–D. However, the crystallographic B-factor for this carbonyl oxygen (22.15) is not particularly high nor is it much larger than that of the carbonyl carbon (21.83). Meanwhile, on the opposite side of that peptide bond’s plane, the amide nitrogen from residue 43 is relatively well superimposed in the NMR ensemble and MD trajectory. The motion of the peptide plane appears to pivot around the amide nitrogen and proton. However, in the crystallographic structure, the B-factor (21.47) is barely lower than that of the carbonyl atoms.

**Figure 2:**
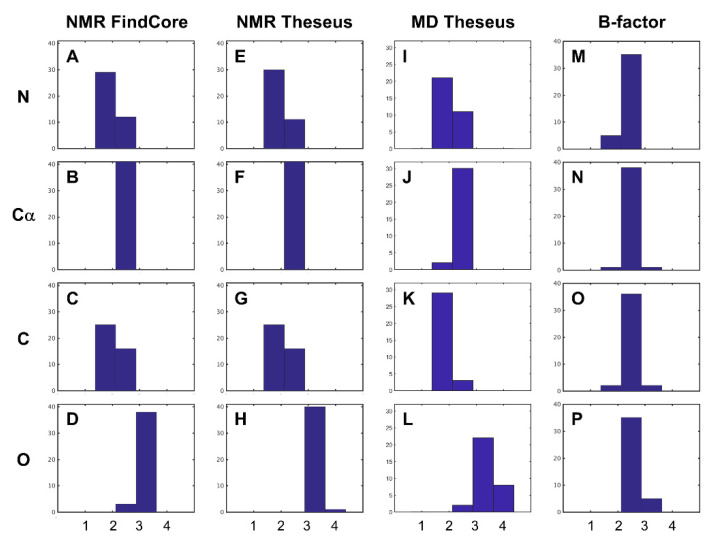
Distribution of average ranks of coordinate uncertainties, variances, and B-factors of backbone heavy atoms. As described in the main text, atoms in each residue are ranked by (**A**–**D**) coordinate uncertainty of FindCore superimposed NMR ensembles, (**E**–**H**) THESEUS superimposed NMR structures, coordinate variances of (**I**–**L**) THESEUS superimposed MD trajectories and (**M**–**P**) B-factors. For each structure, an average rank is calculated for each backbone heavy atom type: (first row) amide N, (second row) Cα, (third row) carbonyl C, and (fourth row) carbonyl O. For superimposed NMR ensembles (columns one and two) and MD trajectories (column three), a clear pattern is visible: average ranks for amide nitrogen atoms and carbonyl carbon atoms are often lower than average ranks for Cα atoms. The average ranks for carbonyl oxygen atoms are usually higher. When backbone heavy atoms are ranked by a B-factor, however, the average ranks for all backbone heavy atoms typically are between 2–3. The average ranks plotted in this figure are tabulated in Tables S2–S5, Supplementary Materials.

Application of Friedman’s test [36] to coordinate uncertainties (Figure 2, first two columns), ranked from lowest to highest on a per-residue basis, of NMR structures, yielded results that confirmed what was observed in the development of the Expanded Findcore method [22]. For almost all NMR ensembles considered, whether superimposed using FindCore or THESEUS, the average rank of the carbonyl oxygen (O) atoms was higher than the average ranks of the amide nitrogen (N), Cα, and carbonyl carbon (C’) atoms. In many structures, the average rank of C’ and N atoms was lower than the average rank of the Cα atoms. Average ranks (averaged on a per-structure basis) of backbone heavy atoms in THESEUS superimposed MD trajectories (Figure 2, third column) were also higher for O atoms and lower for C’ and N atoms. When analyzing crystallographic B-factors, however, average ranks did not generally vary much with the atom type (Figure 2, fourth column).

**Figure 3:**
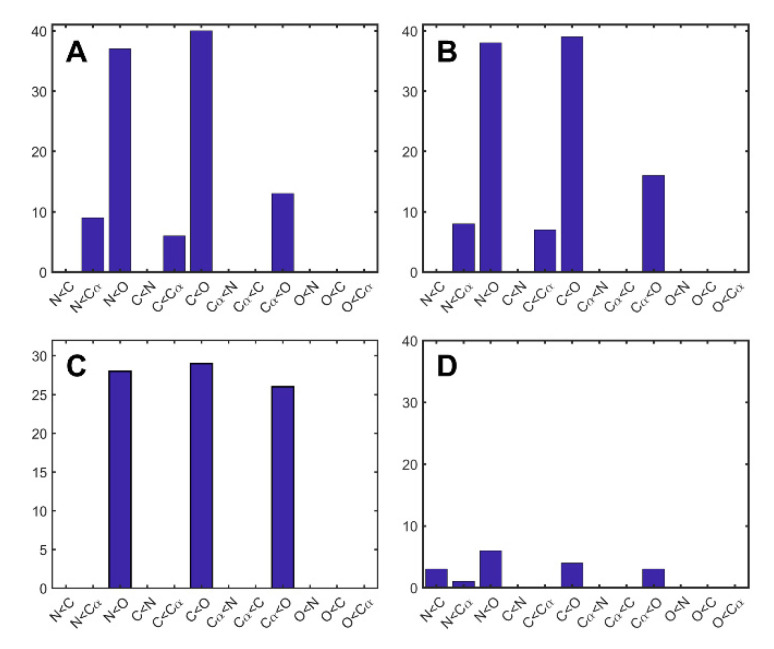
Results of Friedman’s Test and subsequent multiple comparisons analysis. A bar, associated with a comparison X < Y, that is n units high, indicates that, in n structures, the assessed measure of coordinate variability is significantly lower for atom type X than for atom type Y. e.g., in panel A, the bar associated with C < O being 39 units high indicates that in 39 NMR ensembles, the coordinate uncertainties (calculated using FindCore superimpositions) for carbonyl carbons are significantly less (according to Friedman’s test) than those for carbonyl oxygens. Mean ranks are considered significantly different if they differ by more than three standard deviations. Assessed measures of coordinate variability are (**A**) coordinate uncertainties in FindCore superimposed NMR ensembles, (**B**) coordinate uncertainties in THESEUS superimposed NMR ensembles, (**C**) coordinate uncertainties in THESEUS superimposed MD trajectories, and (**D**) crystallographic B-factors. Note that, in almost all superimposed NMR ensembles (independent of superimposition method), as well as in almost all THESEUS superimposed MD trajectories, amide nitrogen and carbonyl carbons have significantly lower coordinate uncertainties than carbonyl oxygens. However, only a small number of crystallographic structures have any significant results using the Friedman’s test to compare B-factors of different atom types.

Multiple comparisons subsequent to Friedman’s test (Figure 3) indicated that, for NMR ensembles and MD trajectories, the coordinate uncertainties and, respectively, variances (as ranked on a per-residue basis) for O atoms were significantly higher than the coordinate uncertainties or variances for N and C’ atoms in almost all ensembles or trajectories explored. In many superimposed NMR ensembles, coordinate uncertainties for O atoms were also significantly higher than coordinate uncertainties for Cα atoms, and, in a few superimposed NMR ensembles, coordinate uncertainties for Cα atoms were higher than those for N and C’ atoms. In most superimposed MD trajectories, coordinate uncertainties for O atoms were also significantly higher than coordinate uncertainties for Cα atoms, but coordinate uncertainties for Cα atoms were not significantly higher than those for N and C’ atoms. However, only a few crystal structures showed any significant differences in coordinate uncertainties between atom types.

Unlike, in the case of superimposed NMR ensembles and MD trajectories, where the coordinate uncertainties or variances of backbone heavy atoms in a residue had a tendency to be lowest for N and C’ atoms and highest for O atoms, no such persistent pattern existed for crystallographic B factors. On the other hand, the pattern in coordinate variances in superimposed MD structures persisted across MD trajectories ran using different forcefields (AMBER99SB vs. OPLS) as well as temperatures (100 K vs. room temperature) and did not depend on whether the SeMET residues found in the crystal structures used to seed MD calculations were replaced with MET residues or not.

That carbonyl oxygens possess a significant tendency to have higher coordinate variances in THESEUS superimposed MD ensembles, as well as having higher coordinate uncertainties across FindCore superimposed NMR “ensembles” indicates the pattern of coordinate uncertainties observed in NMR-derived structures is not solely an artifact of the superimposition method (THESEUS vs. FindCore), not a particular force field used (AMBER and OPLS in MD simulations, CNS [37,38], and XPLOR-NIH [39] in NMR refinement), nor the particular characteristics of an NMR-based structural determination (e.g., a lack of experimentally derived restraints on carbonyl oxygen atoms). The persistence of the tendency for carbonyl oxygens to have higher coordinate variability between ensembles explored via MD simulation and NMR-derived “ensembles”, which typically consist of models resulting from replicated, simulated, annealing calculations, indicates that this tendency is not solely an artifact of the structure sampling scheme used in NMR calculations. It may be the case that NMR structures not refined using CNS or XPLOR-NIH do not generally have carbonyl oxygens with high relative coordinate uncertainties. The one unrefined structure (1XPV) analyzed in this study did have carbonyl oxygens with high relative coordinate uncertainties.

One possible explanation of the high relative carbonyl oxygen uncertainties in superimposed NMR structures and variances in MD trajectories is that forcefields do not adequately restrain the positions of carbonyl oxygens. Carbonyl oxygen atoms are known to favorably interact with aromatic rings via n-π* interactions [40,41] and also participate in hydrogen bonding, whose representation in classical forcefields is often deficient [42]. Hydrogen bonding is important in stabilizing the protein tertiary structure [43], and carbonyl oxygen atoms in regions of a secondary structure typically participate in hydrogen bonds.

**Figure 4:**
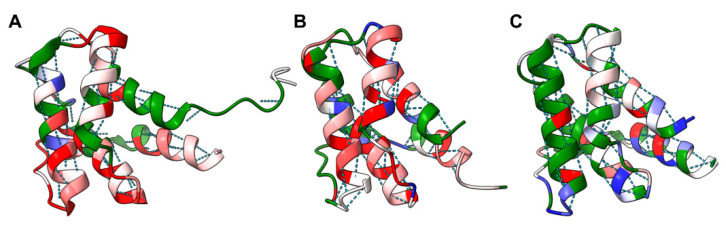
F-scores comparing UniProt ID Q8ZRJ2 backbone heavy atom coordinate uncertainties and variances. (**A**) The first model in the NMR “ensemble” 2JN8, (**B**) final snapshot of the MD trajectory seeded with 2ES9 (replacing Se-MET residues with MET residues, ran at 300 K with the AMBER 99SB forcefield), and (**C**) crystallographic structure, PDB ID 2ES9, each colored on a per residue bases by the F-score described in the Materials and Methods section (Equation (1)). Red indicates an F-score greater than 10 (relative uncertainty, variance, or B-factor of carbonyl oxygen coordinates quite high), white an F-score equal to 1 and blue and F-score less than 0.1. Green indicates residues for which the carbonyl oxygen coordinate uncertainty, variance, or B-factor for the carbonyl oxygen was actually less than the uncertainty, variance, or B-factor for the corresponding amide nitrogen. Dotted lines indicate hydrogen bonds: carbonyl oxygen atoms with high relative coordinate uncertainties and variances occur in both a hydrogen-bonded secondary structure as well as in loop regions. Some helical regions, likely endowed with extra restraints in the NMR-based structure determination process, do have slightly fewer carbonyl oxygens with high relative coordinate uncertainty as compared with the MD trajectory, illustrating the potential importance of hydrogen bonding in “fixing” the position of carbonyl oxygen atoms with high relative coordinate variances. By comparison, the crystallographic structure, PDB ID 2ES9, has relatively few carbonyl oxygens with high relative B-factors as indicated by the relative dearth of red in panel (**C**).

Figure 4 shows that carbonyl oxygen atoms with relatively high coordinate uncertainties in NMR structures and with relatively high coordinate variances in MD trajectories occur in carbonyl oxygen atoms participating in intramolecular hydrogen bonding as well as those which are only hydrogen bonded to solvent. Nevertheless, some carbonyl oxygen atoms in a secondary structure have relatively greater coordinate variances across MD trajectories than in NMR structures. As NMR-based structure calculations typically involve additional restraints on hydrogen bonding atoms (based on H/D exchange data and/or secondary structure as established based on resonance assignments), it may be the case that MD simulations could benefit from better representation of hydrogen bonding [42] and other non-covalent interactions [41] in MD forcefields.

In addition to potentially inadequately representing quantum mechanical phenomena such as hydrogen bonding and n-π* interactions, many force fields strongly penalize any deviation of a peptide bond from planarity. In particular, requiring peptide bonds to remain planar may cause more complex motions of the amide backbone to be represented by simple rocking motions along an axis near the N–C bond axis but angled slightly toward the Cα. This motional model, by placing carbonyl oxygens furthest from the axis of motion (and Cα atoms second furthest), inappropriately represents them as being most mobile. Deficiencies in representing hydrogen bonding in force fields [42] may also be problematic when such deficiencies result in insufficient restraints on carbonyl oxygen positions. Hydrogen bonds that are important in stabilizing protein tertiary structure [43], may represent important restraints in a carbonyl oxygen position across MD trajectories just as they are in NMR-based structural determination.

It is possible that the pattern of coordinate uncertainties and variances observed, respectively, in superimposed NMR and MD ensembles actually represents internal motions of peptide bond units in proteins in the solution state. Carbonyl oxygen atoms, branching off from the main polypeptide chain, may have enhanced thermal motion relative to backbone atoms on the main chain. In fact, other atoms branching off from the main chain, including Cβ atoms and even amide protons, tend to have significantly more coordinate uncertainty in superimposed NMR ensembles and coordinate variance across superimposed MD trajectories than amide nitrogen or carbonyl carbon atoms (Figures S1–S, Supplementary Materials). However, more crystallographic structures have significantly higher Cβ B-factors, as compared to amide nitrogen B-factors by Friedman’s test, than higher carbonyl oxygen B-factors as compared to amide nitrogen B-factors.

It is often assumed that crystallographic B-factors correlate well with internal flexibility. The absence of a persistent pattern in the B-factors for backbone atoms suggests that any such pattern observed in MD trajectories and NMR “ensembles” is an artifact. However, even in an ideal case where Crystallographic B-factors arise entirely from static and dynamic disorder, these B-factors reflect protein dynamics in the crystalline state and not in the solution state [44]. Moreover, previous studies have not only shown that NMR coordinate uncertainties correlate well to coordination variances in MD trajectories but also have demonstrated that crystallization has a “flattening” effect on protein flexibility [25]. Additionally, since Debye-Waller theory attributes any reduction in diffraction pattern intensities relative to those expected given a static protein structure to localize harmonic motion, other processes that reduce diffraction pattern intensities may result in over-estimation or even under-estimation of protein flexibility [45]. Relatedly, values obtained for B-factors are dependent on the refinement techniques used in interpreting X-ray data [25].

Nevertheless, the patterns described in this paper as well as the relatively high correlations between the statistical coordinate uncertainties derived from NMR and the putatively physical coordinate variances across MD ensembles may very well indicate deficiencies common to all force fields. Fully exploring the pervasiveness of the patterns described in this paper necessitates MD simulations and analysis of NMR structures beyond the systems studied here. However, the analysis presented in this paper identifies that coordinate variances/uncertainties from at least some MD trajectories and NMR ensembles have properties not found in B-factors. This divergence between B-factors and coordinate variances potentially indicates that there remain critical concerns in force field development. Future studies of MD trajectories will hopefully reveal which potentially inaccurate aspects of force fields, such as the requirement that peptide bonds remain planar and inadequacies in the representation of non-covalent interactions, such as hydrogen bonding as well as solvent/protein interactions, need the most adjustment. Addressing such deficiencies in force field construction can result in better descriptions of protein structure and, hence, facilitate the accurate prediction of protein dynamics, structure, and folding pathways.

## 3. Materials and Methods

The NMR and crystallographic structures analyzed in this study consisted of all (41) NMR structures and all but one (40) crystallographic structure listed in the “community resource” described by Everett et al. [35], which also outlines standardized methods used by the NESG for solving crystallographic and NMR structures. All but one of the NMR structures (1XPV) analyzed here were refined using CNS [37,38] and/or XPLOR-NIH [39]. MD simulations were performed on a randomly selected set of 12 targets from the community resource, using the conditions indicated in Table S1, Supplementary Materials. Most simulations used the OPLS [46] forcefield, but several simulations were performed with the AMBER99SB [47,48] forcefield as well.

MD simulations were initiated using crystallographic structures retrieved from the Protein Data Bank (PDB, [49]) with the identifications (IDs) listed in Table S1, Supplementary Materials. Simulations were prepared with Schrodinger’s Maestro GUI made available as part of the Desmond [50] software package (which also ran MD simulations), using Na^+^ or Cl^−^ ions to achieve electrical neutrality and the TIP4PEW water model. In order to avoid artifacts due to truncation of the simulated constructs and facilitate parameterization in AMBER99SB, the terminal amino acid residues present in the coordinate sets obtained from the PDB were capped. Simulations ran for up to 36 ns (following default relaxation/minimization protocols), with snapshots recorded every 14.4 ps (up to 2500 snapshots). Re-parameterization of each simulation to use the AMBER99SB force field was performed using Desmond’s Viparr utility. Most simulations were run at room temperature (generally defined for each protein by the temperature at which NMR experiments used to solve the protein’s structure were performed. For all proteins in this study the temperature was very nearly 300 K in order to mimic the conditions in both the NMR tube and during (room temperature) crystallization. Some simulations were also performed at 100 K to mimic conditions obtained during cryo-cooled x-ray diffraction experiments. Simulations were ran both with and without substituting methionine (MET) for the seleno-methionine (SeMET) residues found in crystallographic structures. Dangling ends of protein chains absent from the crystallographic coordinates deposited in the PDB were not filled in computationally but rather were omitted from each simulation.

Initial parsing and visualization of each trajectory were performed using VMD [51]. A simple trajectory rescuer was used prior to initial parsing in VMD for simulations that turned into hung processes. Reformatting was completed for the multi-structural PDB file output from VMD into a multi-model format suitable for further analysis. THESEUS [30] superimposed MD trajectories prior to a coordinate variance calculation and the MATLAB [52] implementation of the FindCore Toolbox superimposed NMR ensembles. Calculation of coordinate uncertainties (calculated as coordinate variances) from FindCore superimposed NMR ensembles used the FindCore Toolbox and calculation of coordinate uncertainties and variances from THESEUS superimposed NMR ensembles and MD trajectories was also performed in MATLAB.

Friedman’s test [36] is a non-parametric analog of ANOVA with repeated measures used here to compare whether coordinate uncertainties, variances, and B-factors are significantly different for different atom types. Application of Friedman’s test proceeded as follows. For each residue in each structure, backbone heavy atom coordinate uncertainties, coordinate variances, or B-factors (depending on the analysis performed) were ranked (from 1–4). For each structure, the resulting ranks were tabulated with columns (treatments) corresponding to a heavy atom type (amide N, Cα, carbonyl carbon, and carbonyl oxygen) and one row (block) for each residue, and the resulting table was subjected to Friedman’s test, which compared column averages (average rank by heavy atom type, averaged on a per-structure basis). MATLAB scripts tabulated B-factor and coordinate variance/uncertainty data for analysis via Friedman’s test and subsequent multiple comparisons, which were also performed in MATLAB. MATLAB was also used to calculate an F-score measuring the relative uncertainties, variances, or B-factors of carbonyl oxygen atoms in a given residue (Equation (1)):

(1) $$F={\left(u\left(O\right)-u\left(N\right)\right)}^{2}/{\left(u\left(C\prime \right)-u\left(N\right)\right)}^{2},$$

where u(.) denotes the coordinate uncertainty, variance, or B-factor of the given atom and O, N, and C′ are the carbonyl oxygen, amide nitrogen, and carbonyl carbon atoms, respectively.’

## Data Availability

Input files used to run the MD simulations analyzed in this paper, the resulting (superimposed) MD trajectories, and all the scripts used to perform the analyses reported here are all archived on Zenodo, doi:10.5281/zenodo.4323630.

## Supplementary Materials

The following are available online: Table S1: Parameters/Input for MD Simulations. Table S2: Average Ranks of Backbone Atom B-Factors for Crystallographic Structures. Table S3: Average Ranks of Backbone Atom Coordinate Uncertainties for Theseus Superimposed NMR “Ensembles”. Table S4: Average Ranks of Backbone Atom Coordinate Uncertainties for FindCore Superimposed NMR “Ensembles”. Table S5: MD Simulation Results. Table S6: Average Ranks of N, C’, Cα, and Cβ B-Factors for Crystallographic Structures. Table S7: Average Ranks of N, C’, Cα, and Cβ Coordinate Uncertainties for Theseus Superimposed NMR “Ensembles”. Table S8: Average Ranks of N, C’, Cα, and Cβ Coordinate Uncertainties for FindCore Superimposed NMR “Ensembles”. Table S9: Average Ranks of N, C’, Cα, and Cβ Coordinate Variances in Theseus Superimposed MD Trajectories. Table S10: Average Ranks of N, C’, Cα, and H Coordinate Uncertainties for Theseus Superimposed NMR “Ensembles”. Table S11: Average Ranks of N, C’, Cα, and H Coordinate Uncertainties for FindCore Superimposed NMR “Ensembles”. Table S12: Average Ranks of N, C’, Cα, and H Coordinate Variances in Theseus Superimposed MD Trajectories. Figure S1: Distribution of average ranks of coordinate uncertainties, variances, and B-factors of N, Cα, carbonyl C, and Cβ atoms. Figure S2: Results of Friedman’s Test and subsequent multiple comparisons analysis with Cβ atoms. Figure S3: Distribution of average ranks of coordinate uncertainties, variances, and B-factors of N, Cα, carbonyl C, and Cβ atoms. Figure S4: Results of Friedman’s Test and subsequent multiple comparisons analysis with amide H atoms.

## Sample Availability

Samples of the compounds are not available from the authors.
