# Supplementary material for: Patterns in Protein Flexibility: A Comparison of NMR “Ensembles”, MD Trajectories, and Crystallographic B-Factors
Source: Molecules. 2021 Mar 9;26(5):1484. doi: 10.3390/molecules26051484 (PMC7967184; doi:10.3390/molecules26051484)
Supplement: Supplementary file 1 [file molecules-26-01484-s001.pdf]

molecules

MDPI

# Supplementary Tables and Figures

**Table S1.** Parameters/Input for MD Simulations.

| *ID* Uniprot | PDB | MSE or MET | pH | Temperature (K) | Ionic Strength (M) | Forcefield |
|---|---|---|---|---|---|---|
| **P20700** | 3JTO | MET | 7 | 298 | 0.100 | OPLS |
| **Q12906** | 3P1X | MET | 6.5 | 298 | 0.100 | OPLS |
| **Q5FJ43** | 3Q69 | MSE | 7 | 293 | 0.200 | OPLS |
| **Q9Y547** | 1TVG | MET | 6.5 | 298 | 0.100 | OPLS |
| **P65294** | 3FIF | MSE | 5 | 293 | 0.100 | OPLS |
| **Q24NW5** | 3LYW | MSE | 9 | 293 | 0.230 | OPLS |
| **Q01826** | 3NZL | MET | 6.5 | 298 | 0.100 | OPLS |
| **P65294** | 3FIF | MET | 6.5 | 293 | 0.100 | OPLS |
| **P74795** | 3C4S | MET | 5.5 | 100 | 0.135 | AMBER |
| **P74795** | 3C4S | MET | 5.5 | 300 | 0.135 | AMBER |
| **P74795** | 3C4S | MET | 5.5 | 100 | 0.135 | OPLS |
| **P74795** | 3C4S | MET | 5.5 | 300 | 0.135 | OPLS |
| **P74795** | 3C4S | MSE | 6.5 | 100 | 0.135 | OPLS |
| **P74795** | 3C4S | MSE | 6.5 | 300 | 0.135 | OPLS |
| **Q7VV99** | 3CPK | MET | 6.5 | 100 | 0.135 | AMBER |
| **Q7VV99** | 3CPK | MET | 6.5 | 300 | 0.135 | AMBER |
| **Q7VV99** | 3CPK | MET | 6.5 | 100 | 0.135 | OPLS |
| **Q7VV99** | 3CPK | MET | 6.5 | 300 | 0.135 | OPLS |
| **Q7VV99** | 3CPK | MSE | 7 | 100 | 0.135 | OPLS |
| **Q7VV99** | 3CPK | MSE | 7 | 300 | 0.135 | OPLS |
| **Q8KFZ1** | 3E0H | MET | 6.5 | 100 | 0.235 | AMBER |
| **Q8KFZ1** | 3E0H | MET | 6.5 | 300 | 0.235 | AMBER |
| **Q8KFZ1** | 3E0H | MET | 6.5 | 100 | 0.235 | OPLS |
| **Q8KFZ1** | 3E0H | MET | 6.5 | 300 | 0.235 | OPLS |
| **Q8KFZ1** | 3E0H | MSE | 4.1 | 100 | 0.235 | OPLS |
| **Q8KFZ1** | 3E0H | MSE | 4.1 | 300 | 0.235 | OPLS |
| **Q8ZRJ2** | 2ES9 | MET | 6.5 | 100 | 0.100 | AMBER |
| **Q8ZRJ2** | 2ES9 | MET | 6.5 | 300 | 0.100 | AMBER |
| **Q8ZRJ2** | 2ES9 | MET | 6.5 | 100 | 0.100 | OPLS |
| **Q8ZRJ2** | 2ES9 | MET | 6.5 | 300 | 0.100 | OPLS |
| **Q8ZRJ2** | 2ES9 | MSE | 6.5 | 100 | 0.100 | OPLS |
| **Q8ZRJ2** | 2ES9 | MSE | 6.5 | 300 | 0.100 | OPLS |

Table S1 collates Uniprot and PDB IDs of structures used to seed MD (Molecular Dynamics) simulations, whether seleno-methionine residues have been replaced with methionine residues (MSE or MET), the forcefield used for MD simulations and the pH, temperature and ionic strength used in each simulation. In general, the pH used in each simulation matched the pH at which the seed structures were obtained. While all simulations were seeded with crystal structures, the ionic strength was that used in NMR-based structure determination. Some "room temperature" simulations were performed at 300 K rather than the exact temperature used in NMR (Nuclear Magnetic Resonance) experiments or crystallization. For some crystal structures calculated using data obtained from cryogenically cooled crystals, simulations were performed at two temperatures, "room temperature" and 100K. The pattern in coordinate variances reported in this paper persists independently of parameters including choice of forcefield, temperature and whether selenomethionines were replaced by methionines.

**Table S2.** Average Ranks of Backbone Atom B-Factors for Crystallographic Structures.

| *ID* | | *Average Ranks* | | | | |
|---|---|---|---|---|---|---|
| **Uniprot** | **PDB** | **N** | **C'** | **Ca** | **O** | **Std. Error** |
| **Q31818** | 3BHP | 2.11 | 2.70 | 2.74 | 2.44 | 0.172 |
| **P74795** | 3C4S | 1.83 | 2.68 | 2.89 | 2.60 | 0.171 |
| **Q7VV99** | 3CPK | 2.23 | 2.58 | 2.38 | 2.80 | 0.094 |
| **Q39VC5** | 3CWI | 1.86 | 2.93 | 2.72 | 2.49 | 0.153 |
| **Q6LYF9** | 3E0E | 2.21 | 2.49 | 2.51 | 2.79 | 0.127 |
| **Q8KFZ1** | 3E0H | 2.34 | 2.60 | 2.72 | 2.34 | 0.100 |
| **E7UZA7** | 2ES7 | 2.40 | 2.52 | 2.39 | 2.69 | 0.104 |
| **Q8ZRJ2** | 2ES9 | 2.26 | 2.73 | 2.62 | 2.38 | 0.116 |
| **Q9Y3C8** | 3EVX | 2.45 | 2.53 | 2.55 | 2.47 | 0.100 |
| **Q99U58** | 2FFM | 2.50 | 2.47 | 2.54 | 2.49 | 0.138 |
| **Q15811** | 3FIA | 2.23 | 2.07 | 2.50 | 3.20 | 0.121 |
| **P65294** | 3FIF | 2.40 | 2.40 | 2.47 | 2.74 | 0.166 |
| **Q8KNE9** | 4FPW | 2.49 | 2.48 | 2.52 | 2.51 | 0.090 |
| **Q9RZE3** | 3GGN | 2.32 | 2.61 | 2.72 | 2.34 | 0.101 |
| **P71066** | 2GSV | 2.21 | 2.59 | 2.62 | 2.58 | 0.152 |
| **Q7U294** | 3GW2 | 2.46 | 2.40 | 2.62 | 2.52 | 0.125 |
| **Q880Y4** | 3H9X | 2.48 | 2.70 | 2.36 | 2.45 | 0.118 |
| **Q8KC80** | 3IBW | 2.45 | 2.51 | 2.52 | 2.52 | 0.015 |
| **Q8U1U6** | 3IDU | 2.42 | 2.54 | 2.44 | 2.60 | 0.026 |
| **P50833** | 2IM8 | 2.41 | 2.51 | 2.74 | 2.33 | 0.115 |
| **Q251Q8** | 3IPF | 1.97 | 2.99 | 2.40 | 2.64 | 0.119 |
| **P20700** | 3JT0 | 2.39 | 2.53 | 2.58 | 2.50 | 0.101 |
| **B2D8H3** | 3K63 | 2.46 | 2.54 | 2.43 | 2.56 | 0.056 |
| **E3YVT8** | 3LD7 | 2.34 | 2.25 | 2.41 | 3.00 | 0.095 |
| **Q6N882** | 3LMO | 2.23 | 2.68 | 2.67 | 2.43 | 0.132 |
| **Q24NW5** | 3LYW | 2.21 | 2.55 | 2.53 | 2.70 | 0.138 |
| **Q2S6C5** | 3MA5 | 2.48 | 2.63 | 2.45 | 2.45 | 0.083 |
| **P15056** | 3NY5 | 2.39 | 2.57 | 2.57 | 2.47 | 0.049 |
| **Q01826** | 3NZL | 1.97 | 2.12 | 2.78 | 3.13 | 0.136 |
| **Q9AAR9** | 2OOQ | 2.13 | 2.76 | 2.46 | 2.65 | 0.120 |
| **Q97RM2** | 3OBH | 2.37 | 2.71 | 2.32 | 2.60 | 0.136 |
| **Q55544** | 3OSJ | 2.39 | 2.55 | 2.47 | 2.58 | 0.037 |
| **Q481E4** | 2OTA | 2.20 | 2.64 | 2.62 | 2.54 | 0.144 |
| **Q12906** | 3P1X | 2.42 | 2.55 | 2.47 | 2.56 | 0.018 |
| **P95883** | 2QOO | 2.33 | 2.28 | 2.45 | 2.94 | 0.116 |
| **Q5FJ43** | 3Q69 | 2.18 | 2.77 | 2.65 | 2.41 | 0.125 |
| **Q8EF26** | 2QTI | 2.53 | 2.14 | 2.12 | 3.22 | 0.142 |
| **P03495** | 2RHK | 2.28 | 2.62 | 2.58 | 2.51 | 0.077 |
| **Q8P6W3** | 1TTZ | 2.28 | 2.74 | 2.57 | 2.41 | 0.146 |
| **Q9Y547** | 1TVG | 2.26 | 2.45 | 2.53 | 2.76 | 0.108 |

Table S2 reports average ranks of backbone heavy atom B factors. Computation of these averages proceeds by ranking backbone heavy atoms on a per-residue basis than averaging the ranks across all residues. MATLAB's [1] friedman [2] and multcompare functions were used to calculate average ranks as well as standard errors and to assist in tabulating the results.

**Table S3.** Average Ranks of Backbone Atom Coordinate Uncertainties for Theseus Superimposed NMR "Ensembles".

| *ID* | | *Average Ranks* | | | | |
|---|---|---|---|---|---|---|
| **Uniprot** | **PDB** | **N** | **C'** | **Ca** | **O** | **Std. Error** |
| **P50833** | 2HFI | 1.96 | 2.05 | 2.83 | 3.16 | 0.116 |
| **P65294** | 2JN0 | 1.84 | 1.92 | 2.62 | 3.62 | 0.183 |
| **Q8ZRJ2** | JN8 | 2.21 | 1.99 | 2.80 | 3.00 | 0.124 |
| **P95883** | 2JPU | 1.94 | 2.08 | 2.74 | 3.24 | 0.114 |
| **P71066** | 2JS1 | 2.04 | 2.08 | 2.68 | 3.21 | 0.144 |
| **Q8EF26** | 2JUW | 2.11 | 2.14 | 2.70 | 3.05 | 0.144 |
| **O31818** | 2JVD | 1.79 | 2.21 | 2.69 | 3.31 | 0.186 |
| **P74795** | 2JZ2 | 1.94 | 2.17 | 2.55 | 3.35 | 0.159 |
| **E7UZA7** | 2JZT | 2.09 | 2.01 | 2.68 | 3.22 | 0.108 |
| **Q9Y3C8** | 2K07 | 1.97 | 2.10 | 2.68 | 3.25 | 0.098 |
| **Q6LYF9** | 2K5V | 1.95 | 1.92 | 2.56 | 3.57 | 0.130 |
| **Q8KFZ1** | 2KCU | 2.18 | 2.13 | 2.73 | 2.96 | 0.100 |
| **Q2S6C5** | 2KCV | 2.02 | 2.18 | 2.62 | 3.18 | 0.130 |
| **Q880Y4** | 2KFP | 2.01 | 2.02 | 2.66 | 3.31 | 0.116 |
| **Q15811** | 2KHN | 2.30 | 1.98 | 2.80 | 2.92 | 0.117 |
| **Q7U294** | 2KKO | 2.19 | 2.27 | 2.66 | 2.89 | 0.124 |
| **P03495** | 2KKZ | 1.87 | 2.13 | 2.64 | 3.37 | 0.112 |
| **Q8U1U6** | 2KL6 | 1.93 | 2.08 | 2.62 | 3.37 | 0.124 |
| **E3YVT8** | 2KPP | 1.84 | 2.13 | 2.46 | 3.56 | 0.121 |
| **P20700** | 2KPW | 2.03 | 2.02 | 2.60 | 3.35 | 0.117 |
| **P62195** | 2KRK | 2.19 | 1.94 | 2.67 | 3.20 | 0.139 |
| **B2D8H3** | 2KRT | 2.00 | 2.03 | 2.59 | 3.38 | 0.117 |
| **Q6N882** | 2KW2 | 1.87 | 2.23 | 2.60 | 3.30 | 0.129 |
| **P15056** | 2L05 | 2.09 | 2.05 | 2.59 | 3.27 | 0.139 |
| **Q55544** | 2L06 | 2.15 | 2.16 | 2.71 | 2.97 | 0.104 |
| **Q01826** | 2L1P | 2.12 | 1.89 | 2.86 | 3.13 | 0.142 |
| **Q12906** | 2L33 | 2.18 | 2.02 | 2.54 | 3.26 | 0.135 |
| **Q97RM2** | 2L3A | 2.22 | 2.00 | 2.51 | 3.27 | 0.143 |
| **Q5FJ43** | 2LFI | 1.99 | 2.23 | 2.48 | 3.30 | 0.117 |
| **Q8KNE9** | 2LUZ | 2.03 | 2.07 | 2.61 | 3.29 | 0.096 |
| **Q99U58** | 1PQX | 1.90 | 2.26 | 2.38 | 3.45 | 0.135 |
| **Q8P6W3** | 1XPV | 2.15 | 2.14 | 2.50 | 3.21 | 0.146 |
| **Q9Y547** | 1XPW | 1.80 | 2.07 | 2.61 | 3.52 | 0.108 |
| **Q9AAR9** | 2JQN | 1.98 | 1.99 | 2.60 | 3.42 | 0.120 |
| **Q481E4** | 2JR2 | 2.17 | 2.04 | 2.84 | 2.95 | 0.148 |
| **Q7VV99** | 2K2E | 2.27 | 1.96 | 2.62 | 3.15 | 0.103 |
| **Q39VC5** | 2K5P | 1.56 | 2.29 | 2.49 | 3.65 | 0.146 |
| **Q9RZE3** | 2KCZ | 2.05 | 2.11 | 2.58 | 3.26 | 0.104 |
| **Q8KC80** | 2KO1 | 1.92 | 2.23 | 2.44 | 3.41 | 0.138 |
| **Q24NW5** | 2KPU | 1.84 | 2.13 | 2.46 | 3.56 | 0.121 |
| **Q251Q8** | 2KYI | 1.82 | 2.12 | 2.54 | 3.51 | 0.108 |

Table S3 reports average ranks of backbone heavy atom coordinate uncertainties calculated from Theseus superimposions. Computation of these averages proceeds by ranking backbone heavy atom coordinate uncertainties on a per-residue basis than averaging the ranks across all residues. MATLAB's friedman and multcompare functions were used to calculate average ranks as well as standard errors and to assist in tabulating the results.

**Table S4.** Average Ranks of Backbone Atom Coordinate Uncertainties for FindCore Superimposed NMR "Ensembles".

| *ID* | | *Average Ranks* | | | | |
|---|---|---|---|---|---|---|
| **Uniprot** | **PDB** | **N** | **C'** | **Ca** | **O** | **Std. Error** |
| **P50833** | 2HFI | 1.93 | 2.05 | 2.84 | 3.19 | 0.116 |
| **P65294** | 2JN0 | 1.92 | 1.92 | 2.63 | 3.54 | 0.172 |
| **Q8ZRJ2** | JN8 | 2.12 | 2.09 | 2.75 | 3.04 | 0.120 |
| **P95883** | 2JPU | 1.90 | 2.07 | 2.74 | 3.29 | 0.114 |
| **P71066** | 2JS1 | 2.03 | 2.05 | 2.69 | 3.24 | 0.144 |
| **Q8EF26** | 2JUW | 2.17 | 2.03 | 2.78 | 3.02 | 0.143 |
| **O31818** | 2JVD | 1.82 | 2.17 | 2.76 | 3.25 | 0.180 |
| **P74795** | 2JZ2 | 2.06 | 2.13 | 2.61 | 3.20 | 0.154 |
| **E7UZA7** | 2JZT | 2.13 | 2.06 | 2.70 | 3.12 | 0.103 |
| **Q9Y3C8** | 2K07 | 2.07 | 2.06 | 2.67 | 3.19 | 0.096 |
| **Q6LYF9** | 2K5V | 2.31 | 2.29 | 2.53 | 2.88 | 0.048 |
| **Q8KFZ1** | 2KCU | 2.23 | 2.12 | 2.73 | 2.91 | 0.098 |
| **Q2S6C5** | 2KCV | 2.04 | 2.18 | 2.62 | 3.17 | 0.129 |
| **Q880Y4** | 2KFP | 2.01 | 2.02 | 2.78 | 3.19 | 0.115 |
| **Q15811** | 2KHN | 2.09 | 2.12 | 2.76 | 3.03 | 0.108 |
| **Q7U294** | 2KKO | 2.20 | 2.26 | 2.68 | 2.86 | 0.124 |
| **P03495** | 2KKZ | 2.17 | 2.25 | 2.61 | 2.97 | 0.066 |
| **Q8U1U6** | 2KL6 | 2.38 | 2.42 | 2.53 | 2.68 | 0.025 |
| **E3YVT8** | 2KPP | 2.05 | 2.07 | 2.50 | 3.38 | 0.112 |
| **P20700** | 2KPW | 2.00 | 2.03 | 2.57 | 3.39 | 0.115 |
| **P62195** | 2KRK | 2.08 | 1.97 | 2.66 | 3.29 | 0.136 |
| **B2D8H3** | 2KRT | 2.01 | 2.02 | 2.61 | 3.36 | 0.117 |
| **Q6N882** | 2KW2 | 1.87 | 2.21 | 2.62 | 3.30 | 0.127 |
| **P15056** | 2L05 | 2.36 | 2.33 | 2.54 | 2.77 | 0.052 |
| **Q55544** | 2L06 | 2.12 | 2.17 | 2.71 | 2.99 | 0.099 |
| **Q01826** | 2L1P | 2.11 | 1.87 | 2.86 | 3.15 | 0.141 |
| **Q12906** | 2L33 | 2.08 | 2.05 | 2.56 | 3.31 | 0.129 |
| **Q97RM2** | 2L3A | 2.18 | 2.01 | 2.52 | 3.29 | 0.142 |
| **Q5FJ43** | 2LFI | 2.03 | 2.20 | 2.54 | 3.22 | 0.112 |
| **Q8KNE9** | 2LUZ | 2.02 | 2.07 | 2.62 | 3.29 | 0.096 |
| **Q99U58** | 1PQX | 1.89 | 2.26 | 2.43 | 3.42 | 0.134 |
| **Q8P6W3** | 1XPV | 2.28 | 2.09 | 2.51 | 3.12 | 0.146 |
| **Q9Y547** | 1XPW | 1.80 | 2.07 | 2.60 | 3.52 | 0.108 |
| **Q9AAR9** | 2JQN | 2.01 | 1.97 | 2.62 | 3.40 | 0.120 |
| **Q481E4** | 2JR2 | 2.19 | 2.00 | 2.81 | 3.00 | 0.148 |
| **Q7VV99** | 2K2E | 2.23 | 2.02 | 2.59 | 3.16 | 0.098 |
| **Q39VC5** | 2K5P | 1.76 | 2.19 | 2.53 | 3.51 | 0.137 |
| **Q9RZE3** | 2KCZ | 2.09 | 2.16 | 2.62 | 3.13 | 0.098 |
| **Q8KC80** | 2KO1 | 1.90 | 2.23 | 2.47 | 3.41 | 0.138 |
| **Q24NW5** | 2KPU | 2.07 | 1.92 | 2.68 | 3.33 | 0.132 |
| **Q251Q8** | 2KYI | 1.94 | 2.15 | 2.54 | 3.37 | 0.094 |

Table S4 reports average ranks of backbone heavy atom coordinate uncertainties calculated from FindCore superimpositions. Computation of these averages proceeds by ranking backbone heavy atom coordinate uncertainties on a per-residue basis than averaging the ranks across all residues. MATLAB's friedman and multcompare functions were used to calculate average ranks as well as standard errors and to assist in tabulating the results.

**Table S5.** MD Simulation Results.

| Uniprot ID | MSE or MET | Forcefield | Simulation Length Time (ns) | # Frames | N | C' | Ca | O | Std. Error |
|---|---|---|---|---|---|---|---|---|---|
| **P20700** | MET | OPLS | 13.6 | 939 | 2.17 | 1.98 | 2.43 | 3.41 | 0.124 |
| **Q12906** | MET | OPLS | 15.8 | 1097 | 1.94 | 2.07 | 2.49 | 3.50 | 0.154 |
| **Q5FJ43** | MSE | OPLS | 16.7 | 1157 | 2.19 | 1.88 | 2.38 | 3.55 | 0.126 |
| **Q9Y547** | MET | OPLS | 18.4 | 1271 | 2.10 | 1.86 | 2.40 | 3.65 | 0.111 |
| **P65294** | MSE | OPLS | 25.8 | 1787 | 1.75 | 2.00 | 2.58 | 3.67 | 0.171 |
| **Q24NW5** | MSE | OPLS | 29.3 | 2031 | 1.95 | 2.09 | 2.43 | 3.52 | 0.139 |
| **Q01826** | MET | OPLS | 30.9 | 2144 | 2.04 | 1.92 | 2.53 | 3.51 | 0.151 |
| **P65294** | MET | OPLS | 36.0 | 2500 | 1.88 | 2.00 | 2.62 | 3.50 | 0.183 |
| **P74795** | MET | AMBER | 36.0 | 2500 | 2.53 | 2.23 | 2.75 | 2.49 | 0.171 |
| **P74795** | MET | AMBER | 36.0 | 2500 | 2.46 | 2.28 | 2.68 | 2.58 | 0.171 |
| **P74795** | MET | OPLS | 36.0 | 2500 | 2.18 | 1.72 | 2.14 | 3.96 | 0.171 |
| **P74795** | MET | OPLS | 36.0 | 2500 | 2.23 | 2.05 | 2.54 | 3.18 | 0.171 |
| **P74795** | MSE | OPLS | 36.0 | 2500 | 2.09 | 2.04 | 1.93 | 3.95 | 0.171 |
| **P74795** | MSE | OPLS | 36.0 | 2500 | 2.35 | 2.12 | 2.49 | 3.04 | 0.171 |
| **Q7VV99** | MET | AMBER | 36.0 | 2500 | 2.23 | 2.11 | 2.43 | 3.23 | 0.095 |
| **Q7VV99** | MET | AMBER | 36.0 | 2500 | 2.25 | 2.06 | 2.49 | 3.20 | 0.095 |
| **Q7VV99** | MET | OPLS | 36.0 | 2500 | 2.06 | 2.05 | 2.28 | 3.61 | 0.095 |
| **Q7VV99** | MET | OPLS | 36.0 | 2500 | 2.11 | 2.13 | 2.43 | 3.34 | 0.095 |
| **Q7VV99** | MSE | OPLS | 36.0 | 2500 | 2.11 | 2.05 | 2.26 | 3.58 | 0.095 |
| **Q7VV99** | MSE | OPLS | 36.0 | 2500 | 2.12 | 2.10 | 2.41 | 3.37 | 0.095 |
| **Q8KFZ1** | MET | AMBER | 36.0 | 2500 | 2.10 | 2.02 | 2.45 | 3.43 | 0.101 |
| **Q8KFZ1** | MET | AMBER | 36.0 | 2500 | 2.21 | 1.96 | 2.55 | 3.28 | 0.101 |
| **Q8KFZ1** | MET | OPLS | 36.0 | 2500 | 2.11 | 1.89 | 2.24 | 3.76 | 0.101 |
| **Q8KFZ1** | MET | OPLS | 36.0 | 2500 | 2.07 | 2.03 | 2.41 | 3.48 | 0.101 |
| **Q8KFZ1** | MSE | OPLS | 36.0 | 2500 | 2.17 | 1.93 | 2.12 | 3.78 | 0.101 |
| **Q8KFZ1** | MSE | OPLS | 36.0 | 2500 | 2.03 | 2.00 | 2.41 | 3.57 | 0.101 |
| **Q8ZRJ2** | MET | AMBER | 36.0 | 2500 | 2.06 | 2.05 | 2.55 | 3.34 | 0.117 |
| **Q8ZRJ2** | MET | AMBER | 36.0 | 2500 | 2.12 | 2.02 | 2.68 | 3.18 | 0.117 |
| **Q8ZRJ2** | MET | OPLS | 36.0 | 2500 | 1.93 | 1.95 | 2.40 | 3.72 | 0.117 |
| **Q8ZRJ2** | MET | OPLS | 36.0 | 2500 | 1.84 | 2.10 | 2.52 | 3.55 | 0.117 |
| **Q8ZRJ2** | MSE | OPLS | 36.0 | 2500 | 1.85 | 2.09 | 2.21 | 3.85 | 0.117 |
| **Q8ZRJ2** | MSE | OPLS | 36.0 | 2500 | 1.84 | 2.10 | 2.52 | 3.55 | 0.117 |

Table S5 reports average ranks of backbone heavy atom coordinate variance calculated from Theseus superimposions of MD trajectories with the indicated number of snapshops. Computation of these averages proceeds by ranking backbone heavy atom coordinate uncertainties on a per-residue basis than averaging the ranks across all residues. MATLAB's friedman and multcompare functions were used to calculate average ranks as well as standard errors and to assist in tabulating the results. Simulations are tabulated here in the same order as in Table S1.

**Table S6.** Average Ranks of N, C', Cα and Cβ B-Factors for Crystallographic Structures.

| *ID* | | *Average Ranks* | | | | |
|---|---|---|---|---|---|---|
| **Uniprot** | **PDB** | **N** | **C'** | **Cα** | **Cβ** | **Std. Error** |
| **O31818** | 3BHP | 2.00 | 2.56 | 2.57 | 2.87 | 0.172 |
| **P74795** | 3C4S | 2.48 | 2.60 | 2.55 | 2.38 | 0.138 |
| **Q7VV99** | 3CPK | 2.19 | 2.47 | 2.26 | 3.07 | 0.122 |
| **Q39VC5** | 3CWI | 2.41 | 2.55 | 2.56 | 2.48 | 0.166 |
| **Q6LYF9** | 3E0E | 2.44 | 2.45 | 2.45 | 2.67 | 0.090 |
| **Q8KFZ1** | 3E0H | 2.24 | 2.57 | 2.47 | 2.72 | 0.101 |
| **E7UZA7** | 2ES7 | 2.21 | 2.62 | 2.53 | 2.64 | 0.152 |
| **Q8ZRJ2** | 2ES9 | 2.46 | 2.62 | 2.41 | 2.51 | 0.125 |
| **Q9Y3C8** | 3EVX | 2.53 | 2.37 | 2.69 | 2.41 | 0.118 |
| **Q99U58** | 2FFM | 2.45 | 2.50 | 2.50 | 2.55 | 0.015 |
| **Q15811** | 3FIA | 2.42 | 2.44 | 2.53 | 2.61 | 0.026 |
| **P65294** | 3FIF | 1.68 | 2.63 | 2.55 | 3.14 | 0.171 |
| **Q8KNE9** | 4FPW | 2.22 | 2.51 | 2.33 | 2.95 | 0.115 |
| **Q9RZE3** | 3GGN | 1.99 | 2.37 | 2.97 | 2.67 | 0.119 |
| **P71066** | 2GSV | 2.37 | 2.49 | 2.53 | 2.61 | 0.101 |
| **Q7U294** | 3GW2 | 2.52 | 2.50 | 2.60 | 2.38 | 0.056 |
| **Q880Y4** | 3H9X | 2.33 | 2.35 | 2.37 | 2.96 | 0.095 |
| **Q8KC80** | 3IBW | 2.13 | 2.54 | 2.62 | 2.71 | 0.132 |
| **Q8U1U6** | 3IDU | 2.08 | 2.45 | 2.43 | 3.03 | 0.138 |
| **P50833** | 2IM8 | 2.59 | 2.61 | 2.70 | 2.09 | 0.083 |
| **Q251Q8** | 3IPF | 2.32 | 2.49 | 2.49 | 2.70 | 0.049 |
| **P20700** | 3JT0 | 1.84 | 2.47 | 2.08 | 3.60 | 0.137 |
| **B2D8H3** | 3K63 | 2.40 | 2.57 | 2.74 | 2.29 | 0.094 |
| **E3YVT8** | 3LD7 | 2.17 | 2.45 | 2.77 | 2.61 | 0.120 |
| **Q6N882** | 3LMO | 2.37 | 2.31 | 2.71 | 2.62 | 0.136 |
| **Q24NW5** | 3LYW | 2.41 | 2.51 | 2.58 | 2.50 | 0.037 |
| **Q2S6C5** | 3MA5 | 2.12 | 2.53 | 2.59 | 2.77 | 0.144 |
| **P15056** | 3NY5 | 2.45 | 2.48 | 2.57 | 2.49 | 0.018 |
| **Q01826** | 3NZL | 2.44 | 2.60 | 2.49 | 2.47 | 0.116 |
| **Q9AAR9** | 2OOQ | 2.05 | 2.44 | 2.58 | 2.93 | 0.125 |
| **Q97RM2** | 3OBH | 2.61 | 2.30 | 2.35 | 2.74 | 0.142 |
| **Q55544** | 3OSJ | 2.22 | 2.52 | 2.60 | 2.66 | 0.077 |
| **Q481E4** | 2OTA | 2.29 | 2.61 | 2.78 | 2.33 | 0.146 |
| **Q12906** | 3P1X | 1.74 | 2.58 | 2.67 | 3.01 | 0.153 |
| **P95883** | 2QOO | 2.21 | 2.37 | 2.39 | 3.03 | 0.108 |
| **Q5FJ43** | 3Q69 | 2.15 | 2.39 | 2.49 | 2.97 | 0.127 |
| **Q8EF26** | 2QTI | 2.19 | 2.45 | 2.42 | 2.93 | 0.100 |
| **P03495** | 2RHK | 2.40 | 2.45 | 2.66 | 2.50 | 0.104 |
| **Q8P6W3** | 1TTZ | 2.15 | 2.52 | 2.60 | 2.74 | 0.116 |
| **Q9Y547** | 1TVG | 2.43 | 2.56 | 2.57 | 2.44 | 0.100 |

Table S6 reports average ranks of N, C', Cα and Cβ B factors. Computation of these averages proceeds by ranking backbone heavy atoms on a per-residue basis than averaging the ranks across all residues. MATLAB's friedman and multcompare functions were used to calculate average ranks as well as standard errors and to assist in tabulating the results.

**Table S7.** Average Ranks of N, C', Cα and Cβ Coordinate Uncertainties for Theseus Superimposed NMR "Ensembles".

| *ID* | | *Average Ranks* | | | | |
|---|---|---|---|---|---|---|
| **Uniprot** | **PDB** | **N** | **C'** | **Cα** | **Cβ** | **Std. Error** |
| **P50833** | 2HFI | 1.76 | 2.50 | 2.07 | 3.67 | 0.116 |
| **P65294** | 2JN0 | 1.83 | 2.46 | 2.16 | 3.55 | 0.098 |
| **Q8ZRJ2** | JN8 | 1.98 | 2.47 | 2.14 | 3.41 | 0.130 |
| **P95883** | 2JPU | 2.07 | 2.47 | 2.16 | 3.31 | 0.100 |
| **P71066** | 2JS1 | 1.96 | 2.44 | 2.34 | 3.25 | 0.130 |
| **Q8EF26** | 2JUW | 1.86 | 2.50 | 2.11 | 3.53 | 0.116 |
| **O31818** | 2JVD | 2.13 | 2.49 | 1.97 | 3.41 | 0.117 |
| **P74795** | 2JZ2 | 2.03 | 2.39 | 2.30 | 3.29 | 0.124 |
| **E7UZA7** | 2JZT | 1.87 | 2.46 | 2.29 | 3.39 | 0.112 |
| **Q9Y3C8** | 2K07 | 1.85 | 2.44 | 2.12 | 3.58 | 0.124 |
| **Q6LYF9** | 2K5V | 1.85 | 2.41 | 2.37 | 3.37 | 0.121 |
| **Q8KFZ1** | 2KCU | 1.80 | 2.50 | 2.02 | 3.68 | 0.183 |
| **Q2S6C5** | 2KCV | 1.94 | 2.43 | 2.09 | 3.54 | 0.117 |
| **Q880Y4** | 2KFP | 2.05 | 2.48 | 2.06 | 3.42 | 0.139 |
| **Q15811** | 2KHN | 1.85 | 2.36 | 2.08 | 3.71 | 0.117 |
| **Q7U294** | 2KKO | 1.80 | 2.48 | 2.44 | 3.29 | 0.129 |
| **P03495** | 2KKZ | 2.00 | 2.33 | 2.06 | 3.62 | 0.139 |
| **Q8U1U6** | 2KL6 | 2.05 | 2.50 | 2.14 | 3.32 | 0.104 |
| **E3YVT8** | 2KPP | 1.87 | 2.48 | 1.87 | 3.78 | 0.142 |
| **P20700** | 2KPW | 2.11 | 2.34 | 2.15 | 3.40 | 0.135 |
| **P62195** | 2KRK | 2.15 | 2.28 | 2.13 | 3.44 | 0.143 |
| **B2D8H3** | 2KRT | 1.90 | 2.39 | 2.36 | 3.35 | 0.117 |
| **Q6N882** | 2KW2 | 1.99 | 2.52 | 2.09 | 3.39 | 0.124 |
| **P15056** | 2L05 | 1.97 | 2.47 | 2.16 | 3.41 | 0.096 |
| **Q55544** | 2L06 | 1.89 | 2.30 | 2.40 | 3.42 | 0.135 |
| **Q01826** | 2L1P | 2.03 | 2.42 | 2.32 | 3.23 | 0.146 |
| **Q12906** | 2L33 | 1.76 | 2.44 | 2.17 | 3.64 | 0.108 |
| **Q97RM2** | 2L3A | 1.95 | 2.47 | 2.11 | 3.47 | 0.120 |
| **Q5FJ43** | 2LFI | 1.95 | 2.49 | 2.00 | 3.57 | 0.148 |
| **Q8KNE9** | 2LUZ | 2.13 | 2.41 | 2.03 | 3.44 | 0.103 |
| **Q99U58** | 1PQX | 1.56 | 2.47 | 2.47 | 3.49 | 0.146 |
| **Q8P6W3** | 1XPV | 1.99 | 2.46 | 2.32 | 3.23 | 0.104 |
| **Q9Y547** | 1XPW | 1.93 | 2.40 | 2.41 | 3.26 | 0.138 |
| **Q9AAR9** | 2JQN | 1.76 | 2.49 | 2.15 | 3.60 | 0.114 |
| **Q481E4** | 2JR2 | 1.85 | 2.41 | 2.37 | 3.37 | 0.121 |
| **Q7VV99** | 2K2E | 1.80 | 2.42 | 2.24 | 3.54 | 0.108 |
| **Q39VC5** | 2K5P | 1.93 | 2.49 | 2.24 | 3.35 | 0.144 |
| **Q9RZE3** | 2KCZ | 1.95 | 2.36 | 2.09 | 3.60 | 0.144 |
| **Q8KC80** | 2KO1 | 1.63 | 2.54 | 2.40 | 3.44 | 0.186 |
| **Q24NW5** | 2KPU | 1.85 | 2.36 | 2.24 | 3.55 | 0.159 |
| **Q251Q8** | 2KYI | 1.92 | 2.44 | 2.10 | 3.54 | 0.108 |

Table S7 reports average ranks of N, C', Cα and Cβ coordinate uncertainties calculated from Theseus superimposions. Computation of these averages proceeds by ranking backbone heavy atom coordinate uncertainties on a per-residue basis than averaging the ranks across all residues. MATLAB's friedman and multcompare functions were used to calculate average ranks as well as standard errors and to assist in tabulating the results.

**Table S8.** Average Ranks of N, C', Cα and Cβ Coordinate Uncertainties for FindCore Superimposed NMR "Ensembles".

| *ID* | | *Average Ranks* | | | | |
|---|---|---|---|---|---|---|
| **Uniprot** | **PDB** | **N** | **C'** | **Cα** | **Cβ** | **Std. Error** |
| **P50833** | 2HFI | 1.72 | 2.51 | 2.06 | 3.71 | 0.116 |
| **P65294** | 2JN0 | 1.95 | 2.38 | 2.17 | 3.50 | 0.154 |
| **Q8ZRJ2** | JN8 | 1.97 | 2.46 | 2.08 | 3.49 | 0.103 |
| **P95883** | 2JPU | 1.93 | 2.45 | 2.07 | 3.54 | 0.096 |
| **P71066** | 2JS1 | 1.75 | 2.51 | 2.31 | 3.43 | 0.138 |
| **Q8EF26** | 2JUW | 2.31 | 2.49 | 2.37 | 2.84 | 0.048 |
| **O31818** | 2JVD | 2.11 | 2.48 | 2.13 | 3.29 | 0.098 |
| **P74795** | 2JZ2 | 1.96 | 2.46 | 2.31 | 3.27 | 0.129 |
| **E7UZA7** | 2JZT | 2.04 | 2.47 | 2.30 | 3.19 | 0.098 |
| **Q9Y3C8** | 2K07 | 1.84 | 2.52 | 2.07 | 3.57 | 0.115 |
| **Q6LYF9** | 2K5V | 1.95 | 2.53 | 2.22 | 3.30 | 0.108 |
| **Q8KFZ1** | 2KCU | 1.90 | 2.52 | 1.95 | 3.63 | 0.172 |
| **Q2S6C5** | 2KCV | 2.03 | 2.41 | 2.27 | 3.29 | 0.124 |
| **Q880Y4** | 2KFP | 2.16 | 2.50 | 2.30 | 3.04 | 0.067 |
| **Q15811** | 2KHN | 2.37 | 2.49 | 2.42 | 2.72 | 0.025 |
| **Q7U294** | 2KKO | 1.90 | 2.41 | 2.39 | 3.31 | 0.138 |
| **P03495** | 2KKZ | 2.06 | 2.44 | 2.19 | 3.31 | 0.112 |
| **Q8U1U6** | 2KL6 | 1.94 | 2.42 | 1.96 | 3.69 | 0.132 |
| **E3YVT8** | 2KPP | 1.93 | 2.43 | 2.13 | 3.51 | 0.115 |
| **P20700** | 2KPW | 1.98 | 2.46 | 2.12 | 3.44 | 0.136 |
| **P62195** | 2KRK | 1.84 | 2.36 | 2.09 | 3.71 | 0.117 |
| **B2D8H3** | 2KRT | 1.78 | 2.51 | 2.39 | 3.32 | 0.127 |
| **Q6N882** | 2KW2 | 1.91 | 2.52 | 2.17 | 3.40 | 0.120 |
| **P15056** | 2L05 | 1.92 | 2.44 | 2.23 | 3.41 | 0.094 |
| **Q55544** | 2L06 | 2.31 | 2.44 | 2.33 | 2.92 | 0.052 |
| **Q01826** | 2L1P | 2.01 | 2.53 | 2.15 | 3.30 | 0.099 |
| **Q12906** | 2L33 | 1.84 | 2.51 | 1.89 | 3.77 | 0.141 |
| **Q97RM2** | 2L3A | 2.05 | 2.42 | 2.18 | 3.35 | 0.130 |
| **Q5FJ43** | 2LFI | 2.10 | 2.31 | 2.13 | 3.46 | 0.142 |
| **Q8KNE9** | 2LUZ | 1.98 | 2.44 | 2.29 | 3.29 | 0.112 |
| **Q99U58** | 1PQX | 1.96 | 2.48 | 2.15 | 3.41 | 0.096 |
| **Q8P6W3** | 1XPV | 1.89 | 2.34 | 2.37 | 3.40 | 0.134 |
| **Q9Y547** | 1XPW | 2.15 | 2.40 | 2.22 | 3.23 | 0.146 |
| **Q9AAR9** | 2JQN | 1.72 | 2.50 | 2.16 | 3.62 | 0.114 |
| **Q481E4** | 2JR2 | 2.13 | 2.41 | 2.07 | 3.38 | 0.098 |
| **Q7VV99** | 2K2E | 1.78 | 2.43 | 2.17 | 3.62 | 0.108 |
| **Q39VC5** | 2K5P | 1.96 | 2.47 | 2.11 | 3.46 | 0.120 |
| **Q9RZE3** | 2KCZ | 1.97 | 2.49 | 1.97 | 3.57 | 0.147 |
| **Q8KC80** | 2KO1 | 1.94 | 2.49 | 2.19 | 3.39 | 0.144 |
| **Q24NW5** | 2KPU | 2.01 | 2.46 | 1.99 | 3.54 | 0.143 |
| **Q251Q8** | 2KYI | 1.70 | 2.61 | 2.27 | 3.42 | 0.180 |

Table S8 reports average ranks of N, C', Cα and Cβ coordinate uncertainties calculated from FindCore superimposions. Computation of these averages proceeds by ranking backbone heavy atom coordinate uncertainties on a per-residue basis than averaging the ranks across all residues. MATLAB's friedman and multcompare functions were used to calculate average ranks as well as standard errors and to assist in tabulating the results.

**Table S9.** Average Ranks of N, C', Cα and Cβ Coordinate Variances in Theseus Superimposed MD Trajectories.

| Uniprot ID | MSE or MET | Forcefield | Simulation Length Time (ns) | # Frames | N | C' | Cα | Cβ | Std. Error |
|---|---|---|---|---|---|---|---|---|---|
| **P20700** | MET | OPLS | 13.6 | 939 | 2.39 | 2.53 | 2.21 | 2.88 | 0.171 |
| **Q12906** | MET | OPLS | 15.8 | 1097 | 2.16 | 2.30 | 2.16 | 3.38 | 0.095 |
| **Q5FJ43** | MSE | OPLS | 16.7 | 1157 | 2.09 | 2.36 | 2.27 | 3.28 | 0.095 |
| **Q9Y547** | MET | OPLS | 18.4 | 1271 | 2.11 | 2.34 | 2.22 | 3.34 | 0.095 |
| **P65294** | MSE | OPLS | 25.8 | 1787 | 2.09 | 2.35 | 2.19 | 3.38 | 0.101 |
| **Q24NW5** | MSE | OPLS | 29.3 | 2031 | 2.19 | 2.40 | 2.12 | 3.30 | 0.101 |
| **Q01826** | MET | OPLS | 30.9 | 2144 | 2.17 | 2.28 | 2.05 | 3.50 | 0.101 |
| **P65294** | MET | OPLS | 36.0 | 2500 | 2.23 | 2.17 | 2.06 | 3.53 | 0.101 |
| **P74795** | MET | AMBER | 36.0 | 2500 | 2.03 | 2.34 | 2.17 | 3.45 | 0.101 |
| **P74795** | MET | AMBER | 36.0 | 2500 | 2.07 | 2.35 | 2.20 | 3.38 | 0.101 |
| **P74795** | MET | OPLS | 36.0 | 2500 | 2.01 | 2.46 | 2.15 | 3.38 | 0.117 |
| **P74795** | MET | OPLS | 36.0 | 2500 | 2.42 | 2.51 | 2.23 | 2.84 | 0.171 |
| **P74795** | MSE | OPLS | 36.0 | 2500 | 2.05 | 2.50 | 2.16 | 3.29 | 0.117 |
| **P74795** | MSE | OPLS | 36.0 | 2500 | 1.97 | 2.43 | 2.05 | 3.55 | 0.117 |
| **Q7VV99** | MET | AMBER | 36.0 | 2500 | 1.94 | 2.28 | 2.26 | 3.52 | 0.117 |
| **Q7VV99** | MET | AMBER | 36.0 | 2500 | 1.83 | 2.50 | 2.24 | 3.44 | 0.117 |
| **Q7VV99** | MET | OPLS | 36.0 | 2500 | 1.83 | 2.50 | 2.24 | 3.44 | 0.117 |
| **Q7VV99** | MET | OPLS | 36.0 | 2500 | 2.26 | 2.21 | 1.88 | 3.65 | 0.171 |
| **Q7VV99** | MSE | OPLS | 36.0 | 2500 | 2.16 | 2.00 | 2.23 | 3.61 | 0.171 |
| **Q7VV99** | MSE | OPLS | 36.0 | 2500 | 2.16 | 2.42 | 2.21 | 3.21 | 0.171 |
| **Q8KFZ1** | MET | AMBER | 36.0 | 2500 | 2.18 | 2.33 | 2.28 | 3.21 | 0.171 |
| **Q8KFZ1** | MET | AMBER | 36.0 | 2500 | 2.20 | 2.32 | 2.19 | 3.29 | 0.095 |
| **Q8KFZ1** | MET | OPLS | 36.0 | 2500 | 2.19 | 2.36 | 2.18 | 3.26 | 0.095 |
| **Q8KFZ1** | MET | OPLS | 36.0 | 2500 | 2.13 | 2.30 | 2.17 | 3.40 | 0.095 |
| **Q8KFZ1** | MSE | OPLS | 36.0 | 2500 | 2.39 | 2.53 | 2.21 | 2.88 | 0.171 |
| **Q8KFZ1** | MSE | OPLS | 36.0 | 2500 | 2.16 | 2.30 | 2.16 | 3.38 | 0.095 |
| **Q8ZRJ2** | MET | AMBER | 36.0 | 2500 | 2.09 | 2.36 | 2.27 | 3.28 | 0.095 |
| **Q8ZRJ2** | MET | AMBER | 36.0 | 2500 | 2.11 | 2.34 | 2.22 | 3.34 | 0.095 |
| **Q8ZRJ2** | MET | OPLS | 36.0 | 2500 | 2.09 | 2.35 | 2.19 | 3.38 | 0.101 |
| **Q8ZRJ2** | MET | OPLS | 36.0 | 2500 | 2.19 | 2.40 | 2.12 | 3.30 | 0.101 |
| **Q8ZRJ2** | MSE | OPLS | 36.0 | 2500 | 2.17 | 2.28 | 2.05 | 3.50 | 0.101 |
| **Q8ZRJ2** | MSE | OPLS | 36.0 | 2500 | 2.23 | 2.17 | 2.06 | 3.53 | 0.101 |

Table S9 reports average ranks of N, C', Cα and Cβ coordinate variances calculated from Theseus superimposions of MD trajectories with the indicated number of snapshops. Computation of these averages proceeds by ranking backbone heavy atom coordinate uncertainties on a per-residue basis than averaging the ranks across all residues. MATLAB's friedman and multcompare functions were used to calculate average ranks as well as standard errors and to assist in tabulating the results. Simulations are tabulated here in the same order as in Table S1.

**Table S10.** Average Ranks of N, C', Cα and H Coordinate Uncertainties for Theseus Superimposed NMR "Ensembles".

| *ID* | | *Average Ranks* | | | | |
|---|---|---|---|---|---|---|
| **Uniprot** | **PDB** | **N** | **C'** | **Cα** | **H** | **Std. Error** |
| **P50833** | 2HFI | 2.02 | 3.08 | 2.43 | 2.47 | 0.116 |
| **P65294** | 2JN0 | 2.07 | 2.93 | 2.44 | 2.55 | 0.098 |
| **Q8ZRJ2** | JN8 | 1.90 | 2.81 | 2.20 | 3.09 | 0.130 |
| **P95883** | 2JPU | 2.17 | 2.86 | 2.39 | 2.57 | 0.100 |
| **P71066** | 2JS1 | 2.15 | 2.95 | 2.59 | 2.31 | 0.130 |
| **Q8EF26** | 2JUW | 2.08 | 3.02 | 2.46 | 2.44 | 0.116 |
| **O31818** | 2JVD | 2.20 | 2.87 | 2.18 | 2.75 | 0.117 |
| **P74795** | 2JZ2 | 2.16 | 2.78 | 2.50 | 2.56 | 0.124 |
| **E7UZA7** | 2JZT | 1.96 | 2.93 | 2.51 | 2.61 | 0.112 |
| **Q9Y3C8** | 2K07 | 1.93 | 2.72 | 2.31 | 3.04 | 0.124 |
| **Q6LYF9** | 2K5V | 1.93 | 2.76 | 2.49 | 2.82 | 0.121 |
| **Q8KFZ1** | 2KCU | 1.86 | 2.94 | 2.38 | 2.82 | 0.183 |
| **Q2S6C5** | 2KCV | 1.89 | 2.71 | 2.20 | 3.20 | 0.117 |
| **Q880Y4** | 2KFP | 2.14 | 2.86 | 2.29 | 2.71 | 0.139 |
| **Q15811** | 2KHN | 2.02 | 2.77 | 2.36 | 2.84 | 0.117 |
| **Q7U294** | 2KKO | 2.08 | 2.92 | 2.71 | 2.29 | 0.129 |
| **P03495** | 2KKZ | 2.14 | 2.78 | 2.38 | 2.70 | 0.139 |
| **Q8U1U6** | 2KL6 | 2.17 | 2.86 | 2.42 | 2.54 | 0.104 |
| **E3YVT8** | 2KPP | 2.10 | 3.01 | 2.22 | 2.67 | 0.142 |
| **P20700** | 2KPW | 2.01 | 2.67 | 2.29 | 3.03 | 0.135 |
| **P62195** | 2KRK | 2.15 | 2.72 | 2.34 | 2.79 | 0.143 |
| **B2D8H3** | 2KRT | 2.12 | 2.79 | 2.62 | 2.47 | 0.117 |
| **Q6N882** | 2KW2 | 2.17 | 2.95 | 2.29 | 2.60 | 0.124 |
| **P15056** | 2L05 | 2.05 | 2.77 | 2.32 | 2.85 | 0.096 |
| **Q55544** | 2L06 | 2.02 | 2.66 | 2.63 | 2.69 | 0.135 |
| **Q01826** | 2L1P | 2.13 | 2.67 | 2.49 | 2.72 | 0.146 |
| **Q12906** | 2L33 | 1.85 | 2.85 | 2.41 | 2.90 | 0.108 |
| **Q97RM2** | 2L3A | 1.98 | 2.84 | 2.36 | 2.82 | 0.120 |
| **Q5FJ43** | 2LFI | 2.12 | 2.99 | 2.32 | 2.58 | 0.148 |
| **Q8KNE9** | 2LUZ | 2.21 | 2.79 | 2.30 | 2.70 | 0.103 |
| **Q99U58** | 1PQX | 1.79 | 2.97 | 2.74 | 2.49 | 0.146 |
| **Q8P6W3** | 1XPV | 2.19 | 2.92 | 2.55 | 2.34 | 0.104 |
| **Q9Y547** | 1XPW | 1.92 | 2.61 | 2.57 | 2.90 | 0.138 |
| **Q9AAR9** | 2JQN | 2.10 | 3.06 | 2.47 | 2.36 | 0.114 |
| **Q481E4** | 2JR2 | 1.93 | 2.76 | 2.49 | 2.82 | 0.121 |
| **Q7VV99** | 2K2E | 1.88 | 2.82 | 2.46 | 2.83 | 0.108 |
| **Q39VC5** | 2K5P | 2.14 | 2.99 | 2.46 | 2.41 | 0.144 |
| **Q9RZE3** | 2KCZ | 2.14 | 2.91 | 2.48 | 2.48 | 0.144 |
| **Q8KC80** | 2KO1 | 1.73 | 3.04 | 2.69 | 2.54 | 0.186 |
| **Q24NW5** | 2KPU | 1.89 | 2.70 | 2.58 | 2.83 | 0.159 |
| **Q251Q8** | 2KYI | 2.13 | 2.89 | 2.42 | 2.56 | 0.108 |

Table S10 reports average ranks of N, C', Cα and H coordinate uncertainties calculated from Theseus superimposions. Computation of these averages proceeds by ranking backbone heavy atom coordinate uncertainties on a per-residue basis than averaging the ranks across all residues. MATLAB's friedman and multcompare functions were used to calculate average ranks as well as standard errors and to assist in tabulating the results.

**Table S11.** Average Ranks of N, C', Cα and H Coordinate Uncertainties for FindCore Superimposed NMR "Ensembles".

| *ID* | | *Average Ranks* | | | | |
|---|---|---|---|---|---|---|
| **Uniprot** | **PDB** | **N** | **C'** | **Cα** | **H** | **Std. Error** |
| **P50833** | 2HFI | 1.96 | 3.12 | 2.41 | 2.50 | 0.116 |
| **P65294** | 2JN0 | 1.94 | 2.67 | 2.42 | 2.97 | 0.154 |
| **Q8ZRJ2** | JN8 | 2.14 | 2.87 | 2.39 | 2.60 | 0.103 |
| **P95883** | 2JPU | 2.11 | 2.88 | 2.37 | 2.64 | 0.096 |
| **P71066** | 2JS1 | 1.91 | 2.92 | 2.53 | 2.64 | 0.138 |
| **Q8EF26** | 2JUW | 2.28 | 2.61 | 2.40 | 2.70 | 0.048 |
| **O31818** | 2JVD | 2.18 | 2.83 | 2.36 | 2.63 | 0.098 |
| **P74795** | 2JZ2 | 2.15 | 2.93 | 2.57 | 2.35 | 0.129 |
| **E7UZA7** | 2JZT | 2.20 | 2.85 | 2.53 | 2.42 | 0.098 |
| **Q9Y3C8** | 2K07 | 2.08 | 3.00 | 2.40 | 2.52 | 0.115 |
| **Q6LYF9** | 2K5V | 2.11 | 2.94 | 2.44 | 2.51 | 0.109 |
| **Q8KFZ1** | 2KCU | 1.92 | 2.93 | 2.25 | 2.90 | 0.172 |
| **Q2S6C5** | 2KCV | 2.17 | 2.77 | 2.47 | 2.59 | 0.124 |
| **Q880Y4** | 2KFP | 2.18 | 2.74 | 2.44 | 2.63 | 0.067 |
| **Q15811** | 2KHN | 2.39 | 2.55 | 2.46 | 2.60 | 0.025 |
| **Q7U294** | 2KKO | 1.91 | 2.64 | 2.56 | 2.90 | 0.138 |
| **P03495** | 2KKZ | 2.00 | 2.66 | 2.32 | 3.02 | 0.113 |
| **Q8U1U6** | 2KL6 | 2.04 | 2.73 | 2.15 | 3.08 | 0.132 |
| **E3YVT8** | 2KPP | 1.89 | 2.73 | 2.25 | 3.14 | 0.115 |
| **P20700** | 2KPW | 2.10 | 2.87 | 2.34 | 2.69 | 0.136 |
| **P62195** | 2KRK | 2.02 | 2.76 | 2.35 | 2.87 | 0.117 |
| **B2D8H3** | 2KRT | 2.03 | 2.95 | 2.69 | 2.34 | 0.127 |
| **Q6N882** | 2KW2 | 2.14 | 2.96 | 2.40 | 2.51 | 0.120 |
| **P15056** | 2L05 | 1.98 | 2.77 | 2.43 | 2.83 | 0.094 |
| **Q55544** | 2L06 | 2.36 | 2.60 | 2.45 | 2.59 | 0.052 |
| **Q01826** | 2L1P | 2.16 | 2.91 | 2.40 | 2.53 | 0.099 |
| **Q12906** | 2L33 | 2.11 | 3.04 | 2.20 | 2.64 | 0.141 |
| **Q97RM2** | 2L3A | 1.97 | 2.71 | 2.31 | 3.01 | 0.130 |
| **Q5FJ43** | 2LFI | 2.10 | 2.73 | 2.33 | 2.84 | 0.142 |
| **Q8KNE9** | 2LUZ | 2.14 | 2.83 | 2.53 | 2.50 | 0.112 |
| **Q99U58** | 1PQX | 2.06 | 2.77 | 2.32 | 2.84 | 0.096 |
| **Q8P6W3** | 1XPV | 1.98 | 2.68 | 2.60 | 2.74 | 0.134 |
| **Q9Y547** | 1XPW | 2.22 | 2.60 | 2.35 | 2.83 | 0.146 |
| **Q9AAR9** | 2JQN | 2.11 | 3.09 | 2.50 | 2.29 | 0.114 |
| **Q481E4** | 2JR2 | 2.20 | 2.76 | 2.31 | 2.72 | 0.099 |
| **Q7VV99** | 2K2E | 1.85 | 2.85 | 2.43 | 2.87 | 0.108 |
| **Q39VC5** | 2K5P | 1.99 | 2.85 | 2.34 | 2.81 | 0.120 |
| **Q9RZE3** | 2KCZ | 2.16 | 2.96 | 2.30 | 2.58 | 0.148 |
| **Q8KC80** | 2KO1 | 2.14 | 3.00 | 2.43 | 2.44 | 0.144 |
| **Q24NW5** | 2KPU | 2.17 | 2.96 | 2.35 | 2.52 | 0.143 |
| **Q251Q8** | 2KYI | 1.70 | 3.03 | 2.51 | 2.76 | 0.180 |

Table S11 reports average ranks of N, C', Cα and H coordinate uncertainties calculated from FindCore superimposions. Computation of these averages proceeds by ranking backbone heavy atom coordinate uncertainties on a per-residue basis than averaging the ranks across all residues. MATLAB's friedman and multcompare functions were used to calculate average ranks as well as standard errors and to assist in tabulating the results.

**Table S12.** Average Ranks of N, C', Cα and H Coordinate Variances in Theseus Superimposed MD Trajectories.

| Uniprot ID | MSE or MET | Forcefield | Simulation Length Time (ns) | # Frames | N | C' | Cα | H | Std. Error |
|---|---|---|---|---|---|---|---|---|---|
| **P20700** | MET | OPLS | 13.6 | 939 | 2.39 | 2.61 | 2.32 | 2.68 | 0.171 |
| **Q12906** | MET | OPLS | 15.8 | 1097 | 2.12 | 2.32 | 2.17 | 3.39 | 0.095 |
| **Q5FJ43** | MSE | OPLS | 16.7 | 1157 | 2.10 | 2.57 | 2.35 | 2.98 | 0.095 |
| **Q9Y547** | MET | OPLS | 18.4 | 1271 | 2.06 | 2.50 | 2.30 | 3.13 | 0.095 |
| **P65294** | MSE | OPLS | 25.8 | 1787 | 2.03 | 2.64 | 2.31 | 3.02 | 0.101 |
| **Q24NW5** | MSE | OPLS | 29.3 | 2031 | 2.17 | 2.71 | 2.27 | 2.84 | 0.101 |
| **Q01826** | MET | OPLS | 30.9 | 2144 | 2.09 | 2.28 | 2.03 | 3.60 | 0.101 |
| **P65294** | MET | OPLS | 36.0 | 2500 | 2.16 | 2.16 | 2.06 | 3.63 | 0.101 |
| **P74795** | MET | AMBER | 36.0 | 2500 | 2.03 | 2.56 | 2.31 | 3.10 | 0.101 |
| **P74795** | MET | AMBER | 36.0 | 2500 | 2.00 | 2.59 | 2.34 | 3.07 | 0.101 |
| **P74795** | MET | OPLS | 36.0 | 2500 | 2.11 | 2.80 | 2.35 | 2.74 | 0.117 |
| **P74795** | MET | OPLS | 36.0 | 2500 | 2.42 | 2.68 | 2.39 | 2.51 | 0.171 |
| **P74795** | MSE | OPLS | 36.0 | 2500 | 2.19 | 2.86 | 2.35 | 2.60 | 0.117 |
| **P74795** | MSE | OPLS | 36.0 | 2500 | 1.92 | 2.45 | 2.12 | 3.51 | 0.117 |
| **Q7VV99** | MET | AMBER | 36.0 | 2500 | 1.90 | 2.32 | 2.25 | 3.54 | 0.117 |
| **Q7VV99** | MET | AMBER | 36.0 | 2500 | 1.79 | 2.75 | 2.41 | 3.05 | 0.117 |
| **Q7VV99** | MET | OPLS | 36.0 | 2500 | 1.79 | 2.75 | 2.41 | 3.05 | 0.117 |
| **Q7VV99** | MET | OPLS | 36.0 | 2500 | 2.23 | 2.23 | 1.82 | 3.72 | 0.171 |
| **Q7VV99** | MSE | OPLS | 36.0 | 2500 | 2.12 | 2.02 | 2.16 | 3.70 | 0.171 |
| **Q7VV99** | MSE | OPLS | 36.0 | 2500 | 2.19 | 2.63 | 2.37 | 2.81 | 0.171 |
| **Q8KFZ1** | MET | AMBER | 36.0 | 2500 | 2.25 | 2.67 | 2.37 | 2.72 | 0.171 |
| **Q8KFZ1** | MET | AMBER | 36.0 | 2500 | 2.13 | 2.50 | 2.34 | 3.03 | 0.095 |
| **Q8KFZ1** | MET | OPLS | 36.0 | 2500 | 2.15 | 2.60 | 2.35 | 2.90 | 0.095 |
| **Q8KFZ1** | MET | OPLS | 36.0 | 2500 | 2.09 | 2.36 | 2.17 | 3.38 | 0.095 |
| **Q8KFZ1** | MSE | OPLS | 36.0 | 2500 | 2.39 | 2.61 | 2.32 | 2.68 | 0.171 |
| **Q8KFZ1** | MSE | OPLS | 36.0 | 2500 | 2.12 | 2.32 | 2.17 | 3.39 | 0.095 |
| **Q8ZRJ2** | MET | AMBER | 36.0 | 2500 | 2.10 | 2.57 | 2.35 | 2.98 | 0.095 |
| **Q8ZRJ2** | MET | AMBER | 36.0 | 2500 | 2.06 | 2.50 | 2.30 | 3.13 | 0.095 |
| **Q8ZRJ2** | MET | OPLS | 36.0 | 2500 | 2.03 | 2.64 | 2.31 | 3.02 | 0.101 |
| **Q8ZRJ2** | MET | OPLS | 36.0 | 2500 | 2.17 | 2.71 | 2.27 | 2.84 | 0.101 |
| **Q8ZRJ2** | MSE | OPLS | 36.0 | 2500 | 2.09 | 2.28 | 2.03 | 3.60 | 0.101 |
| **Q8ZRJ2** | MSE | OPLS | 36.0 | 2500 | 2.16 | 2.16 | 2.06 | 3.63 | 0.101 |

Table S12 reports average ranks of N, C', Cα and H coordinate variances calculated from Theseus superimposions of MD trajectories with the indicated number of snapshops. Computation of these averages proceeds by ranking backbone heavy atom coordinate uncertainties on a per-residue basis than averaging the ranks across all residues. MATLAB's friedman and multcompare functions were used to calculate average ranks as well as standard errors and to assist in tabulating the results. Simulations are tabulated here in the same order as in Table S1.

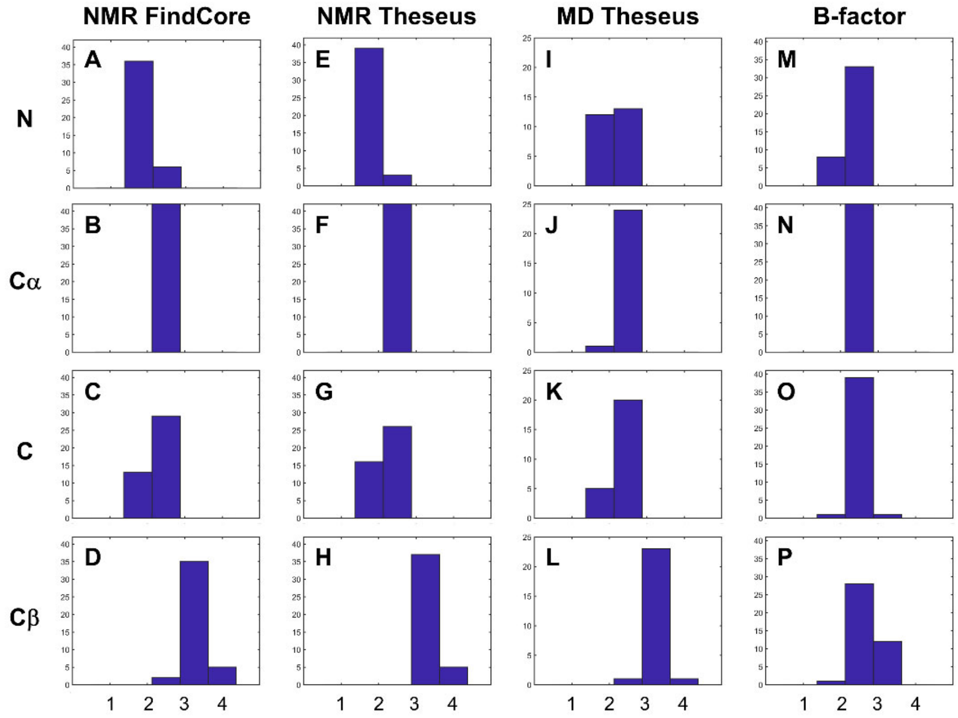


**Figure S1.** Distribution of average ranks of coordinate uncertainties, variances and B-factors of N, Cα, carbonyl C and Cβ atoms. As described in the main text, atoms in each residue are ranked by (**A**–**D**) coordinate uncertainty of FindCore superimposed NMR ensembles, (**E**–**H**) THESEUS superimposed NMR structures, coordinate variances of (**I**–**L**) THESEUS superimposed MD trajectories and (**M**–**P**) B-factors. For each structure, an average rank is calculated for each backbone heavy atom type: (first row) amide N, (second row) Cα, (third row) carbonyl C and (fourth row) Cβ. For superimposed NMR ensembles (columns one and two) and MD trajectories (column three) a clear pattern is visible: average ranks for amide nitrogen atoms and carbonyl carbon atoms are often lower than average ranks for Cα atoms; average ranks for Cβ atoms are usually higher. When backbone heavy atoms are ranked by B-factor, the average ranks for all backbone heavy atoms typically are between 2-3, although Cβ atoms tend to have higher ranks than N, Cα or carbonyl C atoms.

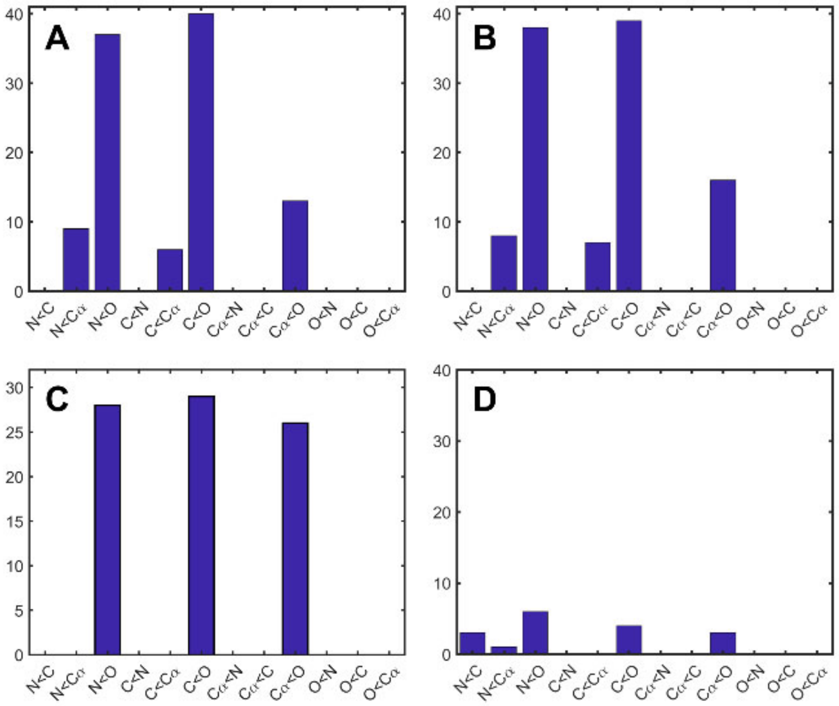


**Figure S2.** Results of Friedman's Test and subsequent multiple comparisons analysis with Cβ atoms. A bar, associated with a comparison X < Y, that is n units high, indicates that in n structures, the assessed measure of coordinate variability is significantly lower for atom type X than for atom type Y. E.g. in panel A, the bar associated with Cα < Cβ being 39 units high indicates that in 39 NMR ensembles, the coordinate uncertainties (calculated using FindCore superimpositions) for Cα atoms are significantly less (according to Friedman's test) than those for Cβ atoms. Mean ranks are considered significantly different if they differ by more than three standard deviations. Assessed measures of coordinate variability are (**A**) coordinate uncertainties in FindCore superimposed NMR ensembles, (**B**) coordinate uncertainties in THESEUS superimposed NMR ensembles, (**C**) Coordinate uncertainties in THESEUS superimposed MD trajectories and (**D**) crystallographic B-factors. Note that in almost all superimposed NMR ensembles (independent of superimposition method), as well as in almost all THESEUS superimposed MD trajectories, amide nitrogens and carbonyl carbons have significantly lower coordinate uncertainties than Cβ atoms. Only a small number of crystallographic structures have any significant results using the Friedman's test to compare B-factors of different atom types, although these numbers are slightly higher than in the comparisons between backbone heavy atoms shown in Fig. 2.

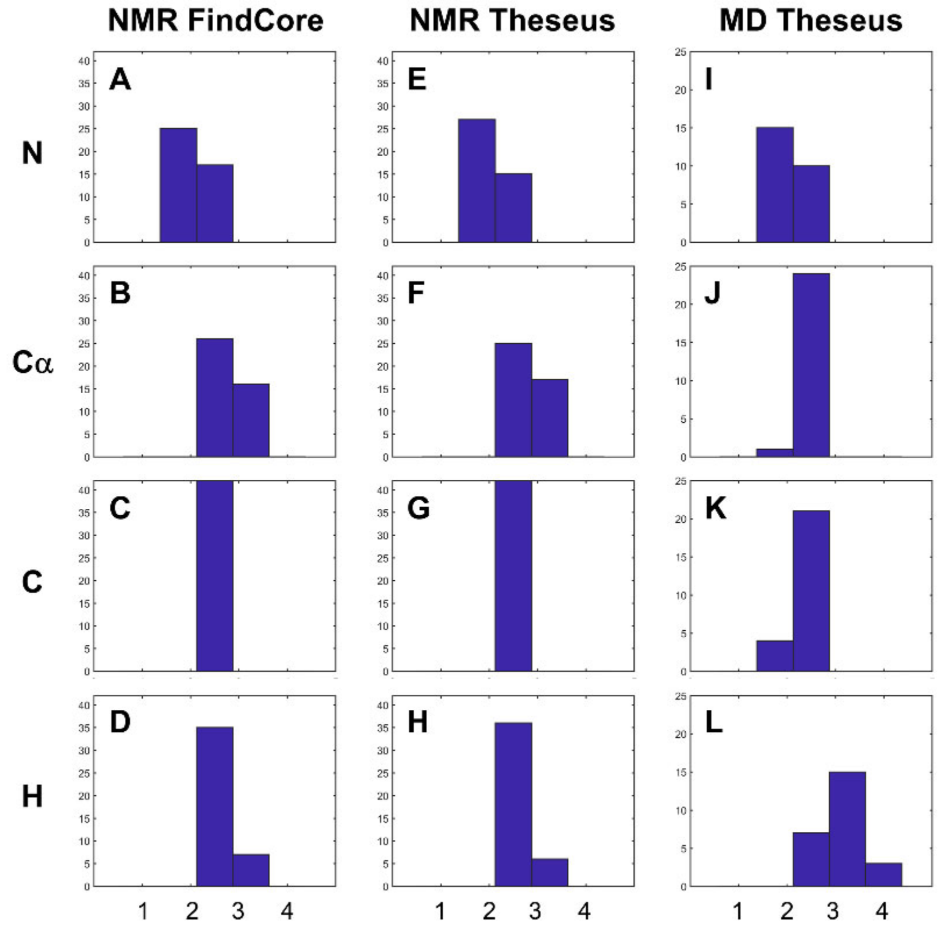


**Figure S3.** Distribution of average ranks of coordinate uncertainties, variances and B-factors of N, Cα, carbonyl C and amide H atoms. As described in the main text, atoms in each residue are ranked by (**A**–**D**) coordinate uncertainty of FindCore superimposed NMR ensembles, (**E**–**H**) THESEUS superimposed NMR structures and coordinate variances of (**I**–**L**) THESEUS superimposed MD trajectories. For each structure, an average rank is calculated for each backbone heavy atom type: (first row) amide N, (second row) Cα, (third row) carbonyl C and (fourth row) H. For superimposed NMR ensembles (columns one and two) and MD trajectories (column three) a clear pattern is visible: average ranks for amide nitrogen atoms and carbonyl carbon atoms are often lower than average ranks for Cα atoms; average ranks for H atoms are usually higher.

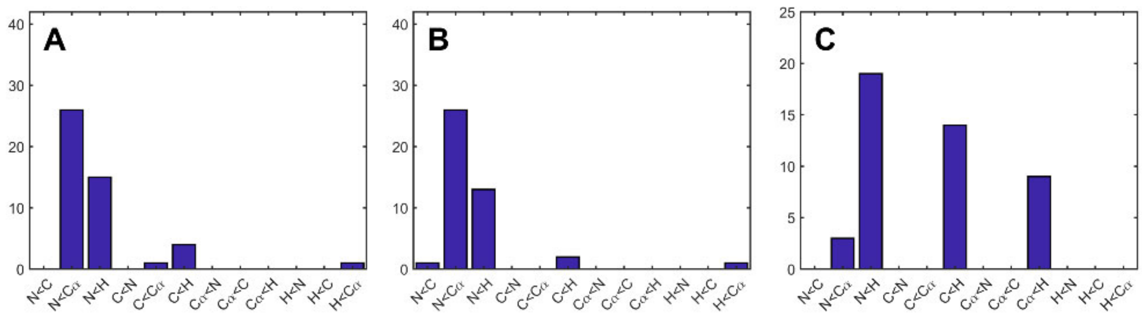


**Figure S4.** Results of Friedman's Test and subsequent multiple comparisons analysis with amide H atoms. A bar, associated with a comparison X < Y, that is n units high, indicates that in n structures, the assessed measure of coordinate variability is significantly lower for atom type X than for atom type Y. E.g. in panel A, the bar associated with Cα < H being 39 units high indicates that in 39 NMR ensembles, the coordinate uncertainties (calculated using FindCore superimpositions) for Cα atoms are significantly less (according to Friedman's test) than those for amide H atoms. Mean ranks are considered significantly different if they differ by more than three standard deviations. Assessed measures of coordinate variability are (**A**) coordinate uncertainties in FindCore superimposed NMR ensembles, (**B**) coordinate uncertainties in THESEUS superimposed NMR ensembles and (**C**) Coordinate uncertainties in THESEUS superimposed MD trajectories. Note that amide H coordinate uncertainties and variances are significantly higher than corresponding backbone heavy atom uncertainties and variances in fewer cases than seen for Cβ and carbonyl O atoms.

## References

1. MATLAB. (2017). version 9.2.0.538062 (R2017a). Natick, Massachusetts: The MathWorks Inc.
2. M Hollander, DA Wolfe, E Chicken, Nonparametric Statistical Methods, 3rd ed., 2015, pp. 1-10.
